# Bispecific T-Cell Redirection versus Chimeric Antigen Receptor (CAR)-T Cells as Approaches to Kill Cancer Cells

**DOI:** 10.3390/antib8030041

**Published:** 2019-07-03

**Authors:** William R. Strohl, Michael Naso

**Affiliations:** 1 BiStro Biotech Consulting, LLC, 1086 Tullo Farm Rd., Bridgewater, NJ 08807, USA; 2 Century Therapeutics, 3675 Market St., Philadelphia, PA 19104, USA

**Keywords:** chimeric antigen receptor, bispecific antibody, T-cell redirection, immune synapse, CD3ε, T cells, NK cells, tumor cell killing, tumor microenvironment

## Abstract

The concepts for T-cell redirecting bispecific antibodies (TRBAs) and chimeric antigen receptor (CAR)-T cells are both at least 30 years old but both platforms are just now coming into age. Two TRBAs and two CAR-T cell products have been approved by major regulatory agencies within the last ten years for the treatment of hematological cancers and an additional 53 TRBAs and 246 CAR cell constructs are in clinical trials today. Two major groups of TRBAs include small, short-half-life bispecific antibodies that include bispecific T-cell engagers (BiTE^®^s) which require continuous dosing and larger, mostly IgG-like bispecific antibodies with extended pharmacokinetics that can be dosed infrequently. Most CAR-T cells today are autologous, although significant strides are being made to develop off-the-shelf, allogeneic CAR-based products. CAR-Ts form a cytolytic synapse with target cells that is very different from the classical immune synapse both physically and mechanistically, whereas the TRBA-induced synapse is similar to the classic immune synapse. Both TRBAs and CAR-T cells are highly efficacious in clinical trials but both also present safety concerns, particularly with cytokine release syndrome and neurotoxicity. New formats and dosing paradigms for TRBAs and CAR-T cells are being developed in efforts to maximize efficacy and minimize toxicity, as well as to optimize use with both solid and hematologic tumors, both of which present significant challenges such as target heterogeneity and the immunosuppressive tumor microenvironment.

## 1. Introduction and History

### 1.1. Historical Context for Immunotherapy

While the concept of immunotherapy goes back to ancient Greek times, the first significant use of prospective immunotherapy was by William B. Coley, in the late nineteenth century [1]. In the early 1880s, an immigrant patient named Fred Stein had a neck tumor that had re-emerged after each attempt to remove it by surgery. Finally, after one surgical procedure, Stein developed an erysipelas infection (*Streptococcus pyogenes*), leading his attending physicians to assume that he would succumb to the infection and die. Stein, however, recovered not only from the infection but also from the cancer. Years later, upon researching Stein’s case, cancer physician William Coley became convinced that the bacterial infection led to a response against the tumor [2,3]. Coley then systematically treated some of his own cancer patients with live bacteria in efforts to stimulate their immune response against the tumors [1,2,3]. These studies yielded variable results but with some clear clinical successes, particularly against sarcomas. Later, Coley used heat-killed pathogens to stimulate the immune system, now known as “Coley’s vaccine” [2,3]. While Coley’s ground-breaking results were hailed by a few, the concept of immune stimulation to treat cancers was not widely accepted and was even scorned by the American Cancer Society for many years [3]. Then, in the late 1990s, over a century after Coley’s initial observations, Bruce Beutler and his colleagues demonstrated that bacterial lipopolysaccharides could agonize toll like receptors (TLRs) [4], which in turn could activate the immune system against cancer [5]. This century-long story has continued to evolve and now has become a major focus in cancer therapy. This review describes two fundamental T-cell-based strategies, as well as variations on those central themes, to harness the power of the immune system to eradicate tumors.

One of the major mechanisms by which cancer cells evade the immune system is via down regulation and loss of their major histocompatibility complex class I (MHC-I) molecules (aka human leukocyte antigens (HLAs)) [6]. Normally, MHC-I-positive tumor cells would be targeted by T-cells with T cell receptors (TCRs) recognizing tumor-specific peptides displayed by the MHC class-I molecules. The recognition and binding of cancer cell surface peptide-loaded MHCs (pMHCs) by TCRs results in the formation of a cytolytic synapse between the T-cell and cancer cell, leading to the directed massive release of cytotoxic proteins such as perforin and granzymes [7], as well as clonal T-cell activation and proliferation [8]. Optimal activation of the T-cells in this and other synaptic interactions requires two signals, the TCR-MHC interaction, known as “Signal 1” and a costimulatory signal (“Signal 2”) through one of several costimulatory receptors on T cells (e.g., CD28, CD137, OX40, CD27, ICOS, GITR) and their cognate ligands (e.g., CD80/86, CD137L, OX40L, CD70, ICOS-L, GITR-L) on the targeted cells or professional antigen-presenting cell (APC) [9]. A third signal, production of immunostimulatory cytokines, helps to drive T cell differentiation and expansion [10]. The MHC-I loss-based mechanism of tumor escape is further complicated by the fact that tumor-cell specific neo-antigens are often “minimal” or difficult to discriminate because they may only be single residue different from their wild-type allele, low affinity or not presented well by MHC-I complex [11].

Loss or downregulation of the MHC-I molecules and absence of strong tumor antigens in cancer cells allow those cells to escape recognition and killing by tumor-infiltrating T-cells which are key components of anti-tumor immunological response [6,12,13]. Additionally, loss of costimulatory molecules (e.g., CD86, CD54) [14], overproduction of checkpoint inhibitory molecules (e.g., PD-1, CTLA4) [15] and tumor production of the tryptophan degrading-enzyme indoleamine 2,3-dioxygenase (IDO), which eliminates tryptophan, a key amino acid required for T-cell proliferation [16], are other examples of mechanisms utilized by tumors to evade cytotoxic T cells.

**Figure 1:**
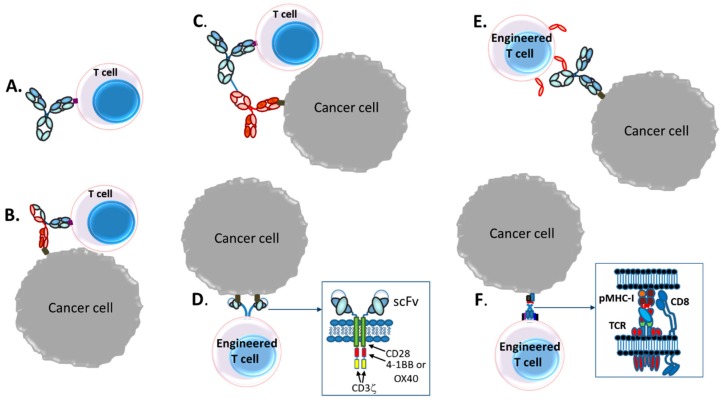
Examples of T-cell based therapeutics in clinical development. (**A**) Inhibition of checkpoint receptors such as PD-1 and CTLA-4 to improve T-cell activity [18]; (**B**) T-cell redirection with bispecific antibodies (TRBAs) in which one binding arm recognizes a tumor antigen and the other binding arm recognizes CD3ε on T-cells [17,19,20]; (**C**) Autologous T-cells activated ex vivo, combined with bispecific antibody conjugates recognizing tumor antigen with one mAb and CD3ε on T cells with the other mAb, followed by re-administration to the patient to kill tumors [21]; (**D**) Genetically engineered autologous chimeric antigen receptor (CAR)-T cells in which an antibody, typically a single chain variable fragment (scFv), fused to intracellular T-cell activation domains such as CD28, 4-1BB, OX40 and CD3ζ, replace the function of the T-cell receptor (TCR), making the T-cells killers of specific antigen-bearing cells [22,23,24]; (**E**) Autologous or allogeneic T-cells or NK cells genetically engineered with FcγRIIIa (CD16a), which, when administered with an anti-tumor monoclonal antibody (mAb) such as the anti-CD20 mAb, rituximab, binds to the Fc of the antibodies and functionally redirects the T-or NK-cells to the tumor to kill the cancer cells [25]; (**F**). Autologous T cells with engineered TCRs.

Today, various therapeutic strategies seek to harness the killing power of T-cells in a TCR functionality-independent manner, bypassing the limitation of HLA-restricted antigen recognition. Two of the most important TCR function-independent T-cell-based therapeutic strategies employed today are T-cell redirecting bispecific antibodies (TRBAs) and chimeric antigen receptor (CAR)-T cells. With TRBAs, the epsilon (ε) domain of cluster of differentiation 3 (CD3), a component of the TCR complex, is targeted with one combining (i.e., binding) domain, while a second binding domain (hence, “bispecific” antibody) binds a tumor cell surface antigen (Figure 1B). These TRBAs function to bring the T-cells and targeted cells into close proximity to form a cytolytic synapse resulting in tumor cell death [17]. In the case of chimeric antigen receptor (CAR)-T cells, a cancer cell surface antigen-targeting antibody fragment, fused to T-cell activating intracellular domains, is expressed as a neo-receptor on the surface of the T-cells (Figure 1D). These tumor antigen-recognizing, “armed” T-cells then will identify, bind and kill the targeted cancer cells. Both of these strategies rely on antibodies to replace the function of the TCR, making them independent of the TCR and its cognate MHC-I/peptide recognition and both can be employed to recognize and target tumor-specific antigens outside the realm of MHC-I-displayed neo-antigen peptides.

Both forms of therapeutic approaches, that is, redirection of T-cells by TRBAs to kill tumor cells and the generation of autologous CAR-T cells from patient T-cells, offer great hope today as next generation antitumor biotherapies [26,27]. These approaches also are being adopted as potential antiviral therapies as well [28,29,30]. As of 20 June 2019, two therapeutics have been approved by major regulatory agencies for each of these T-cell based approaches. In all, there are at least 289 unique T/NK-cell redirected therapeutic candidates, including 61 different TRBAs, 225 unique CAR-Ts and three T/NK cells transduced with CD16a currently being tested in over 320 unique clinical trials (Table 1). Moreover, additional therapeutic approaches utilizing concepts based on these two major T-cell based therapeutic strategies also are being tested in clinical trials (Figure 1).

### 1.2. Brief History of T-Cell Redirecting Bispecific Antibodies

Two fundamental discoveries from the mid-1970s ultimately led to the concept of the TCR function-independent (i.e., as defined by not requiring the recognition and binding of TCR α/β to pMHC) T-cell based therapeutic approaches that are now amongst the most promising paradigms for treating at least some forms of cancer. The first of these is the well-known, Nobel Prize-winning, discovery by Köhler and Milstein [31] of the methods for making and characterizing monoclonal antibodies from hybridomas. The second was the fundamental observation that activated cytotoxic T lymphocytes (CTLs) could function as serial killers of targeted cancer cells [32,33], via formation of an immunological synapse with the targeted cells [34], followed by degranulation and release of cytolytic proteins such as perforin and granzymes [7]. These and other early studies ultimately led to both the development of TRBAs to engage and redirect T-cells to induce serial killing of antigen-specific, targeted cancer cells [20] and to the genetic engineering of autologous T-cells to empower them with cancer cell surface antigen-specific targeting antibody-based receptors (i.e., CARs) fused to T-cell activating domains [35,36,37].

Within ten years of the initial isolation of monoclonal antibodies (mAbs) from immunized mice [31], the first bispecific antibodies were generated using a variety of approaches, including hybrid-hybridomas [38,39], chemical conjugation of both full-length IgGs and of Fabs [40,41,42], formation of bispecific F(ab’)_2_ antibodies using reduction and oxidation of sulfhydryl bonds processes [43] and recombinant approaches to make bispecific antibody fragments [44,45] based on single chain variable fragments (scFvs) [46,47].

Perhaps underappreciated today in the tsunami of T-cell redirecting bispecific antibodies and CAR-T cells, the first concepts and practice of redirecting T cells through binding of one antibody recombining (or binding) site to T cell surface markers to kill tumor cells bound by the other recombining site were laid out in several papers in the 1985–1986 time frame [41,42,43]. The use of the CD3 component of the TCR as the T cell target for redirection was first described shortly thereafter, in 1987 [48].

**Figure 2:**
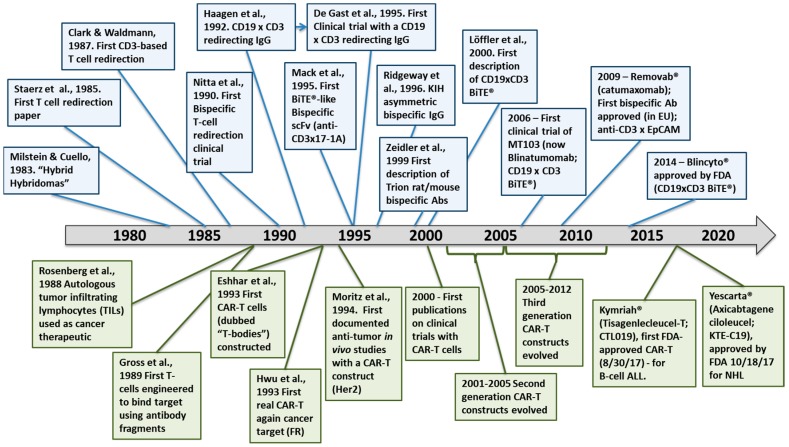
Key milestones in the history of T-cell redirected bispecific antibodies (TRBAs; top) and chimeric antigen receptor (CAR)-T cell (bottom) therapeutics. Specific references cited for TRBAs are: Milstein and Cuello, 1983 [38], Staerz et al., 1985 [41], Clark and Waldmann, 1987 [48], Nitta et al., 1990 [49], Haagen et al., 1992 [50], De Gast et al., 1995 [51], Mack et al., 1995 [45], Ridgeway et al., 1996 [52], Zeidler et al., 1999 [53] and Löffler et al., 2000 [54]. Specific references for CAR-T development cited are: Rosenberg et al., 1988 [55], Gross et al., 1989 [35], Eshhar et al., 1993 [37], Hwu et al., 1993 [56] and Moritz et al., 1994 [57].

Figure 2 lays out a brief history of T-cell redirected bispecific antibodies and CAR-T therapeutics. Several key advances in the 1990s laid the foundation for the wide variety of T-cell redirecting bispecific antibody formats used for clinical stage candidate antibodies today. The first clinical trial in which T-cell redirecting bispecific antibodies were dosed was in 1990, when patients with glioblastoma were treated with an anti-CD3 IgG chemically coupled to an anti-glioma antigen IgG [49]. This was closely followed by the generation [50] and use in clinical studies [51] of an anti-CD19 × anti-CD3 bispecific IgG-like rat/mouse hybrid bispecific antibody for treatment of B-cell lymphomas. This antibody was the first IgG-like T cell redirecting bispecific antibody targeting malignant B cells to be studied in clinical trials [51].

Another significant advance in the late 1980s and 1990s was the discovery of methods to generate single chain variable fragment (scFv) antibody constructs by linking the two domains of an Fv, the variable heavy (V_H_) and variable light (V_L_) domains together using a short flexible linker [46,47], followed by the fusion of two scFvs together via a peptide linker to generate the first bispecific T cell engager (BiTE^®^)-like antibody [45] Figure 2). The first BiTE^®^ targeted the tumor antigen 17-1A on the target cell with one scFv and CD3 on the T-cell with the other scFv arm [45]. The first description of an anti-CD19 × CD3 BiTE^®^ was in 2000 [54]. The final significant advance in the 1990s was the generation of the now well-known asymmetric, heterodimeric Fc platform, “knobs-into-holes” (KIH), by scientists at Genentech [52,58,59] (Figure 2). This platform became the prototype for an entire generation of IgG-like asymmetric bispecific antibodies modified in the C_H_3 domain to allow for heterodimeric antibody formation [60]. After engineering a production cell line with two heavy chains, one with a “knob” or protruding amino acid residue, mutation in the interface region of the C_H_3 domain and the other with a compensating “hole” or small amino acid residue mutation and two light chains, the resultant heterodimer could be formed in four possible HC–LC pairings, in which the desired format is only one of the antibody molecules [59]. This technology was subsequently improved with the use of common LCs to eliminate the “light chain issue,” that is, pairing of the light chains with the correct Fc half [59]. Interestingly, though, in the decade following the Merchant et al. paper [59], very few advances were made in the engineering of bispecific antibodies and most of the activity was focused on just two clinical candidates. Starting in about the 2007–2009 timeframe, however, the interest in developing new bispecific antibody platforms and using these to make TRBAs and other bispecific antibody therapeutics literally exploded, resulting in the development of more than one hundred different new platforms [60,61,62].

The first TRBA and bispecific antibody of any kind, to be approved by a major regulatory agency for commercial use was catumaxomab (trade name Removab^®^), a hybrid mouse-rat IgG-like bispecific antibody targeting CD3ε on T-cells with one arm and the cancer antigen, epithelial cell adhesion molecule (EpCAM), with the other arm [53,63]. Catumaxomab, which appears to have first entered clinical trials around the 2001–2002 timeframe [64], was approved in 2009 by the European Medicines Agency (EMA) as a therapy to treat malignant ascites [65]. However, due to its high immunogenicity rates in humans (being a fully rodent antibody), narrow and rare approved indication (i.e., malignant ascites) and subsequently poor sales, Removab^®^ was not actively marketed past 2014 and was voluntarily discontinued by its sponsor in 2017. Removab^®^ was never approved by the United States Food and Drug Administration (US-FDA).

The second T-cell redirecting antibody to be approved for therapeutic use was blinatumomab (trade name Blincyto^®^), a fragment-based bispecific antibody called a BiTE^®^, in which two single chain variable fragments (scFvs), one targeting the B cell antigen, CD19 and the other CD3ε, were linked together with a short, five residue (G_4_S)_1_ linker, that is,: ((V_L_CD19-(GGGGS)_3_-V_H_CD19)-GGGGS-(V_H_CD3-(GGS)_4_GG-V_L_CD3ε)) [66]. Blinatumomab, which was first known as Micromet MT103 (aka MedImmune MEDI-538), first entered clinical trials in 2006. Blincyto^®^ was approved by the US-FDA in 2014 for treatment of Philadelphia chromosome-negative, B-cell acute lymphoblastic leukemia (ALL), making it the second TRBA to be approved for therapeutic use [67].

From just three TRBAs being studied in clinical trials in the 2008 timeframe (catumaxomab [63,64,65]), blinatumomab [68] and ertumaxomab, a rat/mouse TRBA targeting HER2 [69], there are now 59 unique clinical candidate CD3ε-binding TRBAs either approved by a regulatory agency or being studied in clinical trials today, with another two redirecting NK cells, totaling 61 TRBAs (Table 1).

### 1.3. Brief History of CAR-T Cells

It was clear from studies in the late 1970s that CTLs were capable of serial killing of targeted cancer cells [32,33]. This concept logically led to the idea of utilizing the power of autologous tumor infiltrating lymphocytes (TILs) to treat the tumor from which they were derived [55,70]. For this approach, TILs were harvested from human tumors, expanded ex vivo for four to eight weeks and then were re-administered intravenously along with a dose of interleukin-2 (IL-2) to help stimulate the re-administered lymphocytes [55]. This treatment resulted in regression of metastatic tumors in 60% of patients treated. While these results were preliminary, they clearly demonstrated the potential use of tumor-specific, expanded and activated autologous T-cells in cancer therapy [55].

The use of autologous TILs as therapeutics, however, still suffered from the lack of robust tumor targeting and the ability to control which cells were targeted. The first successful engineering of T-cells with a known and specific artificial binding capability was reported in 1989 (Figure 2), when Gross et al. [35] fused the V_H_ and V_L_ chains of an anti-2,4,6-trinitrophenol (TNP) antibody onto either the Cα-chain or Cβ-chain (i.e., V_H_-Cα/V_L_-Cβ and vice versa) of the TCR to generate artificial, chimeric TCRs. T-cells engineered in this manner were capable of killing TNP-coated target cells in a non-MHC-restricted manner [35]. While this engineered cell construct itself was not a CAR-T cell as we think of it today, it led directly to the formation of first-generation CAR-T cells.

**Figure 3:**
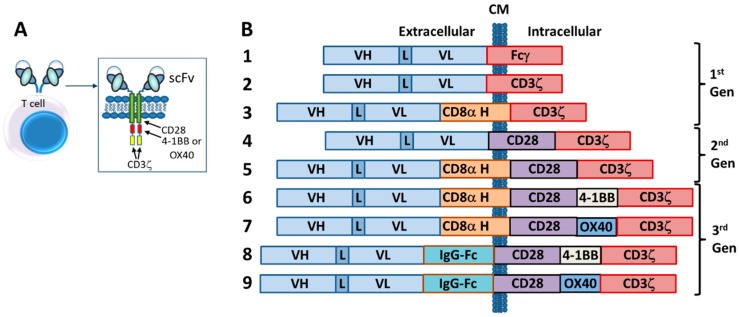
Generations of CAR-T cell therapeutics. Generations of CAR-T cell therapeutics as described in the text. (**A**) Generalized drawing of a CAR-T showing the fusion of the scFv to the transmembrane domain and intracellular activation domains. (**B**) Drawing depicting examples of first generation (1–3), second generation (4, 5) and third generation (6–9) CAR-T constructs as described in the text.

The first CARs, which targeted the hapten TNP, consisted of an scFv (V_L_-linker-V_H_) fused directly to the human Fc receptor γ-chain, replete with its short extracellular domain, transmembrane domain and the immunoreceptor tyrosine activation motifs (ITAMs) [37] (Figure 3). The CD3ζ chain, which is highly similar in sequence and function to the γ-chain, also was used as the intracellular signaling domain in the fusion [37]. These first CAR-T cells were dubbed “T-bodies” denoting the construction of T cells with CARs made up of antibodies [71]. While the concept of CAR-T cells has been around since the early 1990s [37], only in the past decade have the technologies advanced to the point required to turn this into a viable “manufacturable” process. Thus, analogous to the TRBA approach, CAR-Ts are conceptually old but functionally still relatively young and developing [72].

One of the earliest “real” cancer targets for CAR-T cell engineering was a cell surface folate binding protein (later determined to be folate receptor (FR)), implicated as a target in ovarian cancer. A first-generation anti-FR CAR was constructed by fusion of an scFv derived from the anti-FR antibody, MOv18, with Fcγ-chain, as described above [73] (Figure 3). T-cells transduced with this CAR (named *Mov-γ*) killed FR^+^ IGROV-1 ovarian adenocarcinoma cells in vitro [73] and increased the survival of mice implanted with IGROV-1 ovarian adenocarcinoma cells [56]. This construct then was used in one of the early clinical trials of autologous CAR-Ts to treat ovarian cancer patients [74]. Due to the limited first-generation design, treatment with the *Mov-γ* CAR-Ts resulted in the lack of CAR-T persistence, poor trafficking to the tumor site and no reduction in tumor burden for any patient [74]. In the same period, Moritz et al. [57] carried out the first preclinical in vivo studies with a CAR-T cell line targeting HER2 (Figure 2).

The first two reports of clinical trials using CAR-T cells were published in the year 2000 (Figure 2). Mitsuyasu et al. [75] described the treatment of HIV-infected patients with a CAR comprised of the extracellular and transmembrane domains of human CD4 fused with the intracellular domain of the CD3ζ, resulting in a few patients having a transient drop in viral titer. Additionally, Junghans et al. [76] reported the results of a clinical trial in which cancer patients were treated with a CAR against carcinoembryonic antigen (CEA). Additional early clinical efforts using CAR-T cells have been reviewed by Eshhar [77].

Other first-generation CARs incorporated the inert transmembrane domain from CD8 between the scFv and the intracellular signaling γ-chain or CD3ζ chain [78,79] (Figure 3). All of these first-generation CAR-T constructs suffered from the fact that, while they could engage and kill targeted cells in vitro and in in vivo rodent models, they lacked the ability to persist in vivo [22,74]. This is most likely due to the absence of a costimulatory signal (i.e., signal 2), because tumor cells rarely express a costimulatory receptor ligand (e.g., B7, OX40L) [80]. Additionally, the lack of the costimulatory signal can render the T-cells anergic [81] and potentially susceptible to apoptosis [82]. Thus, it was quickly realized that additional signaling would be required to construct biologically active CAR-T cells that would persist in vivo.

Second generation CAR-T cells were designed by adding to the γ-chain or CD3ζ CAR constructs a cytoplasmic signaling domain from a costimulatory receptor, such as CD28 [83,84,85], 4-1BB (CD137) [84] or OX40 (CD134) [84] (Figure 3). These constructs typically resulted in improved production of activating cytokines such as IL-2 and IFN-γ, increased antigen-dependent proliferation in vitro and upregulated apoptotic factors such as Bcl-X_L_ [83,84,85]. Nevertheless, even with second generation CARs, it appeared that T-cell activation was still not complete [80]. Thus, a series of third generation CARs was designed and these are starting to be incorporated into clinical trials today. Third generation CARs combine internal domains for CD28 plus intracellular signaling domains from either OX40 (CD134) [80,86] or 4-1BB (CD137) [86,87] (Figure 3), resulting in cytolytic T cells fortified with both proliferation and survival signals that enhance both their cell killing activity and their persistence in circulation.

Subsequently, it was demonstrated that a longer and more flexible “hinge” region (i.e., extracellular spacer such as regions from IgG-Fc or CD8α) was required for optimal CAR activity [88] (Figure 3) and, over the years since then, significant efforts have been made to optimize both the length and the structural characteristics of the extracellular spacer [89,90,91].

It might seem obvious that the addition of more T-cell activating signals to CARs would result in more robust tumor cell killing. Although certain studies have shown this to be the case, it is still not clear that “more is better” in every case. Various in vitro and in vivo studies have described both improvement and limitations in engineered T-cell function dependent on the design of the CAR [92,93,94,95]. T cell exhaustion and anergy, as well as the often negative influence on the T-cell by the tumor microenvironment, involve a carefully orchestrated series of signals within the T-cell that are poorly understood and not easily accommodated by CAR engineering, as of yet [96]. Similarly, the fine tuning of the molecular architecture of the CAR is also recognized as an area that needs to be improved, as the complicated physiochemical nature of the complete T-cell receptor complex is starting to be revealed [97].

For fourth generation CAR-T cells, new functions have been added beyond the target binding and T-cell activating signals. These most recent approaches include functions such as an inducible caspase-based suicide mechanism to eliminate the CAR-T cells on demand [98], expression and secretion of T-cell activating cytokines [99], the incorporation of trafficking receptors such as CCR2 to help the T-cell home to tumor microenvironments [100] or the use of virus-specific T cells that recognize viral antigens which can be used as “vaccines” to increase the persistence of the CAR-T construct [101,102,103].

Two CAR-T based therapeutics have thus far been approved by major regulatory agencies. Kymriah^®^ (Tisagenlecleucel-T; also known as CTL019), the first CAR-T to be approved for therapeutic use, was approved on August 30, 2017 by the US-FDA for treatment of B-cell ALL [104]. Yescarta^®^ (Axicabtagene ciloleucel; also known as KTE-C19), was approved by the US-FDA on October 18, 2017 for treatment of diffuse large B-cell lymphoma (DLBCL) [105]. There are now at least 223 additional recombinant CAR cell-based candidates being studied in clinical trials today (Table 1).

## 2. T-Cell Synapse and Killing Target Cells

### 2.1. Introduction to Immunological Synapse

The immunological or immune, synapse is a central mechanism of action for lymphocytes to communicate via cell-cell interaction with antigen-presenting cells (APCs), antigen-specific targeted cells and other lymphocytes. In normal T cell biology, small (~5 µm diameter) circulating naïve CD8^+^ T cells find an antigen-presenting cell (APC) and form a synapse with the APC via interaction of clustered TCRs on the surface of T cells with the neo- or non-self peptide antigen-loaded MHC molecules on the surface of the APC [8]. This interaction results in differentiation and activation of the CD8^+^ T-cells over the next 4–5 days into “armed” antigen-specific killer T cells loaded with granules full of the cytolytic proteins, granzyme and perforin [8]. These primed antigen-specific T cells expand and proliferate, increase in diameter to ~10 µm, induce a more sophisticated cytoskeletal system to “load” the cytolytic granules in proximity to the cell membrane [106] and express additional receptors of activation and response [8,107]. Upon locating the cells expressing the non-self or neo- antigen, typically either neoplastic or infected cells, the T-cells form the cytolytic synapse with the target cells and release their cytolytic toxins to kill those cells [107]. Additionally, T-cell membrane lytic factors such as FasL also can act in the synapse to induce apoptosis in the targeted cells [107].

**Figure 4:**
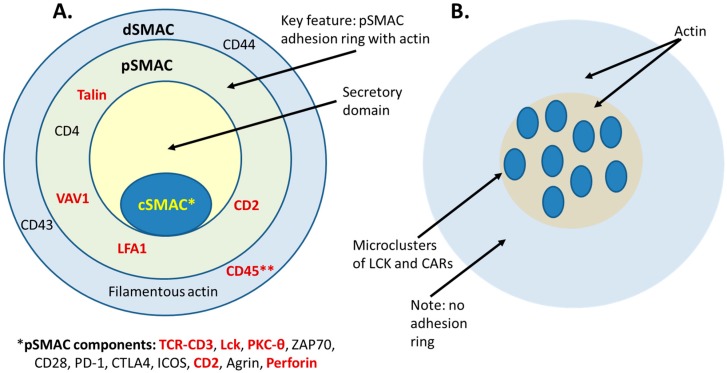
Classical immune synapse as compared with a bispecific T-cell engager (BiTE^®^)-induced synapse and a CAR-T synapse. (**A**) Diagrammatic representation of the immune synapse, adapted and modified from Huppa and Davis [110] and Watanabe et al. [111]. The classical immune synapse forms as a “bullseye” with the center central supramolecular activation cluster (cSMAC) surrounded by the peripheral SMAC (pSMAC) adhesion ring and the distal dSMAC ring. CD3, PKC-θ, perforin, CD28, CTLA4 and Agrin are found in the cSMAC. Additionally, Lck initially accumulates in the cSMAC and then distributes more broadly [110]. A key feature of the immune synapse is exclusion of CD45 from the cSMAC (noted by **). The pSMAC ring includes Talin, LFA1, VAV1 and CD4. LFA-1 is a key synapse stabilizing force in the pSMAC. The dSMAC markers are CD43, CD44, CD45 and filamentous actin. Offne×r et al. [13] compared the synapses formed by an anti-EpCAM × CD3 BiTE^®^ TRBA to those formed by MHC-Her2-peptide/TCR. The markers denoted in red were positioned similarly in both the normal peptide-loaded major histocompatibility complex (pMHC)/TCR synapse and the BiTE-induced synapse [13]. CD45 was found to be excluded from both the BiTE^®^-induced synapse and the control pMHC/TCR synapse [13]. (**B**) A diagrammatic representation of the synapse formed by CAR-T cells, adopted and modified from Davenport et al. [112]. They described the CAR-T/target cell synapse as disorganized, with multifocal clusters containing LCK, no apparent LFA-1 stabilization and the absence of the adhesion ring that helps to define the classical immune synapse [112,113].

The immune synapse, also known as the supramolecular activation cluster (SMAC), is responsible for initiating and completing the cell-cell response between APCs and T cells [8]. The SMAC is formed in three concentric rings, similar to a “bullseye” (Figure 4), with the central SMAC (cSMAC) forming the center ring, encircled by the peripheral SMAC (pSMAC) and the distal SMAC (dSMAC). Each ring has its own special function and structure [8]. The cSMAC contains a concentration of TCRs and the costimulatory molecule CD28 and is responsible for the key T-cell activation signaling events that accompany synapse formation, the pSMAC contains a series of adhesion molecules such as LFA-1 that stabilize the cell-cell interaction and the dSMAC is comprised of filamentous actin that helps to exert a mechanical force on the synapse [8].

There are multiple forms of the immune synapse, each with its own special function. The classical immune synapse, as exemplified by naïve CD4^+^ T cells interacting with APCs, is an antigen recognition synapse. CD8^+^ cells and NK cells can form stimulatory synapses leading to cytokine secretion or alternatively, inhibitory synapses [108]. CD8^+^ T cells and NK cells also can form a cytolytic synapse with target cells leading to killing, which is the basis on which T-cell and NK cell redirected therapies are based.

Cytolytic synapses are very similar to the classic immune synapse but with additional activities to drive target cell killing. These include actin and microtubule guided localization of the lytic proteins [106], signals directing the secretion of cytotoxic proteins such as perforin and granzymes [109] and use of the mechanical forces of the dSMAC to enhance perforin activity and focus the cytotoxic killing in a directional, polarized manner [8,107].

### 2.2. Normal TCR-pMHC Synapses vs. CAR-T and TRBA-Induced Synapses

**Figure 5:**
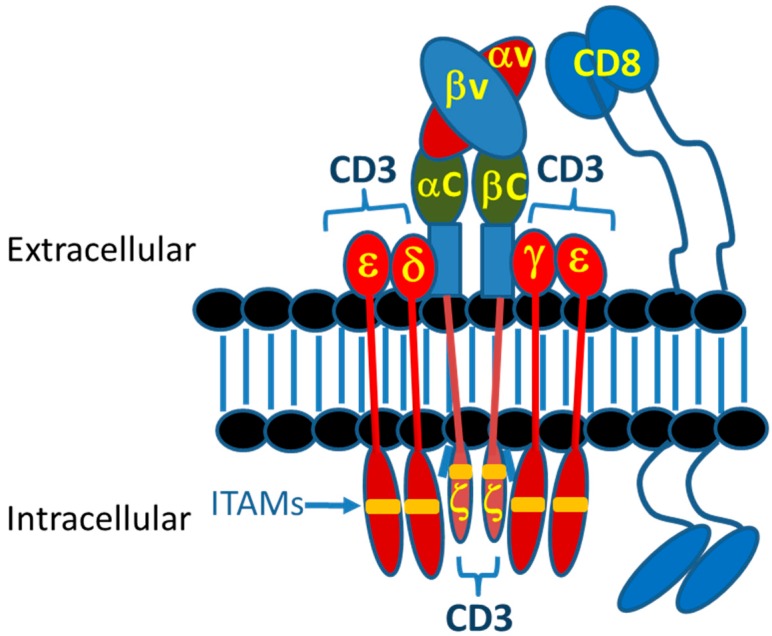
Diagram of the T-cell receptor (TCR) complex. Normal TCR-pMHC interactions are in the range of 1–100 µM, with the inherent avidity of clustered TCRs providing the required attraction [11]. Affinities of the MHC to the presented peptide have a profound influence on the ability of natural T cells to kill and eradicate tumors. When the peptide-MHC affinity was found to be <10 nM as determined in in vitro assays, it was shown that T cells recognizing those pMHC complexes were able to cause tumor rejection [116]. On the other hand, when the peptide-MHC affinity was >100 nM, the tumors relapsed, indicating that the T cells were not capable of killing those tumors cells [116]. Both CAR-T cells and TRBAs function independently of this parameter.

The delicately orchestrated events associated with a TCR complex-MHC interaction signaling into the T cell has been an area of research for some time. The TCR complex is a complicated structure of a TCR α/β or δ/γ heterodimer that, analogous to an antibody, precisely recognizes peptide-MHC (pMHC) complexes. However, the intracellular signals associated with this interaction come from the other members of this complex, namely CD3ε, δ, γ and ζ. These chains are specifically associated with the TCR α/β (δ/γ) heterodimer at the cell surface through ionic bonds made between the transmembrane and hinge/stalk domains [114] (Figure 5). Although previously thought to be just a clustering-driven event that drives downstream signaling through phosphorylation of key residues within ITAMs, it is now recognized that important structural changes during this interaction drive the strength and duration of the downstream events [114]. In addition, the co-receptors CD4 and CD8, both dimers themselves, are required to interact and specifically bind to either class I (CD8) or class II (CD4) MHC in the context of TCR α/β complexes (TCR δ/γ complexes do not require CD4/CD8 co-receptor engagement to function). The intracellular domains of CD4 and CD8 also associate with the Src kinase LCK to provide additional signaling, the function of which is not completely understood [115]. In total, this complicated structure has evolved to control one of the most complex cellular activities within mammals and other organisms. The goals of TRBA and CARs has always been to mimic this complexity as much as possible.

The T cell/target cell synapse is driven by a delicate balance between affinity and receptor-target density, with regard to natural TCR-pMHC interactions, TRBA and CARs. The consequence of these interactions can be influenced by natural regulatory receptors, such as CD45 isoforms, which can naturally down-regulate the signals emanating from the TCR or CARs [117]. Embedded in the cell membrane, CD45, which is a complex, highly differentially spliced molecule of varying extracellular size, can interfere with these synapse-based interactions and prevent downstream signaling [118]. Low-affinity interactions, typical of TCR-pMHC interactions, are very susceptible to the effects of CD45 isoforms, serving as a natural safety mechanism to prevent undesired T cell activation [117]. However, higher affinity interactions or multiple interactions between the TCR and pMHC can overcome these effects [117]. Similar considerations must also be taken into account when designing TRBA and CARs.

**Figure 6:**
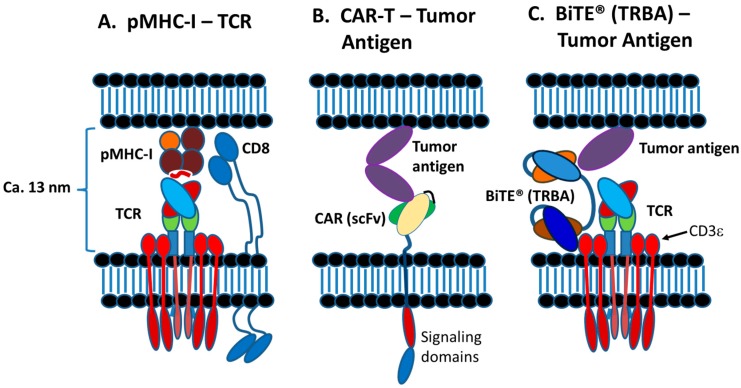
Diagram of the T-cell receptor complex. Diagram of the molecules driving synapse formation in pMHC-1/TCR T-cell/APC interactions, in CAR-T/target cell interactions and in TRBA-induced T-cell/target cell interactions. (**A**) Classic TCR/pMHC-1 type of interaction with a membrane to membrane spacing in the range of 13 nm [119]. (**B**) scFv-based CAR-T cell binding to tumor antigen on target cell. (**C**) BiTE^®^ binding to CD3ε on T-cell and to tumor antigen on target cell to bring the cells into close proximity to form the cytolytic synapse.

The spacing between T cells and APCs during synapse formation has been measured in the range of 5–25 nm [8] and the normal spacing in synapses formed between T cell TCRs and peptide-loaded MHC (pMHC) complexes has been shown to be about 13 nm [119] (Figure 6). Experimentally forced longer distances between the cell membranes decreased the TCR activation and response [119], which is a key issue for both TRBAs and CAR-Ts, as described later.

For natural CTLs, it has been shown that as few as 1–3 peptide-MHC/TCR interactions are required to trigger a cytolytic killing event [107,120,121]. In those cases, however, the elaborate SMAC complex is neither required nor fully formed [121]. Additionally, it has recently been demonstrated with NK cells, another cytolytic lymphocyte, that NK cell lines only produce about 200 perforin-positive granules and a single degranulation event at the cytolytic synapse results in only about 20 granules being released, only about 2–4 of which are actually required to kill a target cell [122]. Thus, the machinery for cytolytic synapse-based killing is exquisitely potent and sensitive to activation.

How do the synapses induced by TRBAs or those formed between CAR-T cells with targeted cells, compare with natural synapses? A study by Baeuerle and colleagues demonstrated that the synapse formed between T-cells and EpCAM^+^ cells, brought together by an anti-EpCAM × CD3ε BiTE^®^ TRBA, was highly similar in its structure and function to normal cytolytic T-cell synapses, including formation of the concentric rings and presence of many of the same protein markers, such as LCK, PKC-θ, LFA-1, VAV1, Talin, CD3, perforin and CD2 [13] (Figure 4). Furthermore, it was demonstrated separately that the TRBA-induced synapse also possessed classical synapse hallmarks [13], including target clustering, ZAP70 translocation and exclusion of the negative regulatory protein, CD45, from the cSMAC [15].

While synapses formed by the function of TRBAs appear to be highly similar to normal MHC/TCR mediated synapses, CAR-T synapses appear to be significantly different from normal T-cell synapses [113]. CAR-T synapses are not highly organized as SMACs but rather, they are disorganized, patchy signaling clusters lacking a defined structure [112]. Additionally, CAR-T synapses do not require LFA-1 for stabilization and do not form the characteristic pSMAC. Thus, it is clear that immune synapses formed by CAR-T cells with their target cells are structurally distinct from both classical immune synapses and those formed by TRBAs (Figure 4). These structural differences between CAR-T synapses and classical pMHC/TCR synapses also result in functional differences [113]. CAR-T cells yield faster proximal signaling and recruit lysosomes to the immune synapse faster than classical synapses, suggesting that they are able to mount a more rapid killer response than TCR-mediated killing [112]. Additionally, they have a significantly faster off-rate, that is, dissolution of the synapse and detachment from the target cell, than found with TCR-driven T cell interactions [112,113,123]. In a time course comparison of TCR-mediated killing versus CAR-T killing after synapse formation, CAR-T signal strength was both greater and ramped up faster than TCR signaling, perforin and granzyme release were faster (peak release within two minutes of initiation for CAR-T vs three min for TCR) and detachment was significantly faster (at five min for CAR-T vs seven min for TCR) [123]. Thus, CAR-Ts appear to kill target cells faster and then move on faster than CTLs. Additionally, it has been demonstrated in vitro that a CAR-T cell can kill a target antigen-positive tumor cell within about 25 minutes of initially recognizing the target antigen [124]. Recently, Xiong et al. [125] demonstrated that the strength of the CAR-T synapse, as measured by quantification of actin and lytic granules, was more predictive of killing effectiveness than either cytokine production of a 4-h killing assay, demonstrating how important the CAR-T synapse is to CAR-T function, even if it is structured significantly different from traditional immune synapses [112].

As noted above, cytolytic TCR/pMHC synapses, TRBA-induced synapses and CAR-T-target cell synapses all result in death of the target cells, typically by perforin and granzyme-induced apoptosis [109], although with CAR-T cells, the FAS/FAS-L axis is also involved [123]. As expected from the differences in structures (Figure 6), however, the TCR-independent synapses formed by TRBAs and CAR-Ts have some unique features as compared with TCR-pMHC synapses. A few of these differences will be highlighted below.

First, both TRBAs and CAR-T cells function independently of TCR/pMHC and thus, do not require expression of MHC receptors on target cells. This was demonstrated in a study in which BiTE^®^s were shown to kill EpCAM-positive, MHC Class-I-negative cell lines, indicating that BiTE^®^s could function to kill target cells in the total absence of the MHC T cell recognition molecules [13]. Second, the affinities of TCRs for pMHCs are typically in the range of 1–100 µM [11], whereas affinities of CARs or TRBAs to their targets are typically below 100 nM and often below 10 nM.

Third, T-cell activation due to interaction via TCR/pMHC is in part governed by the expression of pMHC on the target cells, which are typically found at very low copy number [126]. Targets for TRBAs and CAR-T cells, on the other hand, typically number in the 1000 s to 10,000 s and sometimes even higher. Nevertheless, TRBAs have been shown to elicit T-cell killing even with very low antigen densities on target cells, such as only 200 target molecules/cell [15]. Thus, even at low target densities, at least in some circumstances, TRBAs can still be effective killing agents.

CAR-T cells also appear to be able to kill targeted cells when antigen densities reach levels as low as 200 target molecules/cell [127,128]. In studies directly comparing the activity of TRBAs and analogous CAR-T cells, it appears that the CAR-T cells are more effective at killing targeted cells at low antigen density than was the analogous BiTE^®^s [127,128]. Interestingly, similar to the hierarchical threshold found with TCR signaling [129], the antigen density required to trigger CAR-T killing (100–200 targets/cell) was significantly lower than the antigen density required to trigger CAR-T cytokine release (~5000 targets/cell) [111,128]. Thus, it appears that, similar to TCR responses, there are distinct thresholds in CAR-T cell activation for killing, proliferation and cytokine release, with a full response requiring a density of at least 5000 antigens/cell [128].

On the other hand, in what appears to be very different from TRBA or pMHC/TCR-mediated synapse formation, CAR-T cells may directly contribute to target cell antigen loss resulting in low antigen density. In a very recent study, it was demonstrated that CAR-T cells decreased antigen density on target cells through the mechanism of trogocytosis, a process by which targeted antigens are transferred to the CAR-Ts [130]. This process not only decreased antigen density on the target cells but also once the CAR-T cells obtain the target antigen, they themselves can become targets of CAR-T fratricide, potentially contributing to lack of persistence [130].

Fourth, it was calculated that, for the most potent BiTE^®^ with a fM IC50, “double-digit” (i.e., <100) TRBA-driven cell-cell interactions were required to drive synapse formation [131]. Similarly, with CAR-T cells, it has been estimated that as low as 100 or less CAR-antigen interactions are required to drive synapse formation between CAR-T cells and their target cells [111]. These estimates both are at least a log greater than the number of interactions required than the minimal number of TCR-pMHC interactions required to initiate a synaptic killing event [107,120,121].

Finally, T-cell activation via TCR-pMHC interaction is enhanced by recruitment of CD8 [115] and incorporates “signal 2” costimulatory pathways [9,18]. Conversely, T-cells are negatively regulated by checkpoint interactions such as PD-1/PD-L1 and CTLA4/CD80-86 [18,132]. Both T-cells engaged by TRBAs and CAR-T cells can function independently of signal 2. Nevertheless, the effector function and targeted killing by T-cells engaged by TRBAs can be enhanced by costimulatory molecules such as CD28 and CD137 (4-1-BB) [133]. For CAR-T cells, the CARs themselves are designed with intracellular costimulatory signaling domains from CD28, OX40, ICOS and/or 4-1BB, providing the costimulatory signal upon binding of the CAR to the targeted cells [94,125,134].

Similar to the regulation of CTLs, T-cells engaged by TRBAs [15,135,136,137] and CAR-T cells [138] can be subjected to inhibition by checkpoint pathways such as PD-1/PD-L1 and CTLA4/CD80-86. With this in mind, clinical trials are currently underway combining the treatment of B-cell lymphomas or leukemias with the anti-CD19 BiTE^®^, Blincyto^®^, with anti-PD-1 [139,140] or anti-PD-1 and anti-CTLA4 antibodies [141].

Similarly, anti-PD-1 and/or anti-CTLA4 checkpoint inhibitor co-therapy also is being tested clinically with CAR-T therapeutics in efforts to relieve checkpoint inhibition of the CAR-T cells [142]. In some cases, fourth generation CAR-T cells are being engineered to express antibodies or antibody fragments that can function in an autocrine/paracrine manner to block checkpoint inhibitors such as PD-1 or PD-L1 [143,144,145] or engineer into the CAR-T cells dominant-negative PD-1 that negates the PD-1/PD-L1 inhibitory pathway [138]. In both of these cases, the inhibitory effects of PD-1 were blocked, increasing the effector functions and persistence of the CAR-T cells. In addition, clinical trials with CAR-T cells engineered by knocking out their endogenous PD-1 gene are being run to test their hypothesized improved efficacy [146].

As noted above, while there are significant differences in the mechanisms by which T-cells are activated in the CTL (TCR/pMHC), TRBA or CAR-T cell paradigms, there are also many similarities. These include the formation of synapses by redirected T cells with target cells, ability to function as serial killers, their mechanism of killing, (e.g., directed release of cytolytic proteins such as perforin and granzymes to kill the targeted cells), their regulation via costimulatory and checkpoint inhibitory pathways and their ability to proliferate and secrete cytokines [17].

## 3. T-Cell Redirecting Bispecific Antibodies (TRBAs)

### 3.1. Introduction

Bispecific antibodies are antibodies that have two different types of combining regions (variable domain-based binding sites), which makes them capable of binding two different antigens simultaneously. Of the approximately 858 antibodies either currently in clinical trials or approved by a major regulatory agency (WR Strohl, BiStro Biotech Consulting Antibody and CAR-T Database, last updated 20 June 2019), there are currently 122 unique clinical stage bispecific antibodies, 59 of which are CD3ε-binding, T-cell redirecting bispecific antibodies and two (GT Biopharma GTB-3550 [147] and Affimed AFM13 [148] of which are CD16a (FcγRIIIa) NK-cell redirecting antibodies (Table 2).

**Figure 7:**
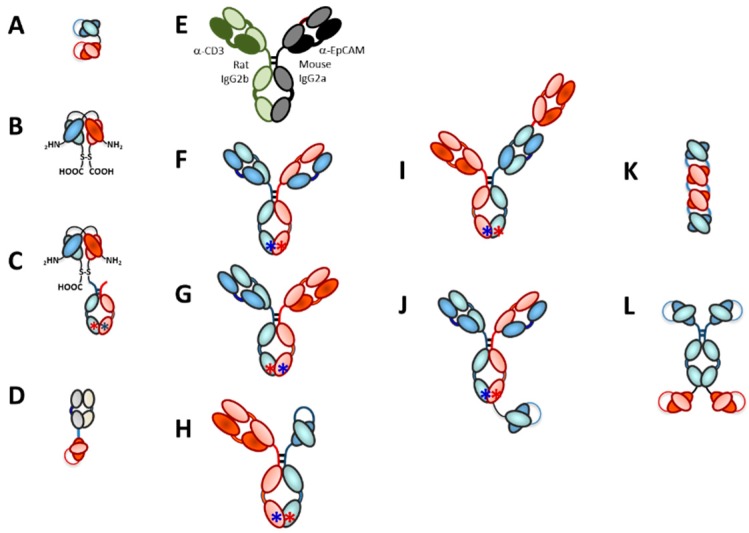
Examples of bispecific antibody platforms used to make clinical stage TRBAs. (**A**) Bispecific T-cell engager (BiTE^®^) [45]; (**B**) dual affinity retargeting (DART^®^) antibody [153]; (**C**) DART^®^-Fc for elongated half-life in vivo [154]; (**D**) TCR fused to scFv called immune-mobilizing monoclonal TCRs against cancer (ImmTAC) [155]; (**E**) mouse/rat hybrid IgG [53]; (**F**) Asymmetric IgG with common LC [59]; (**G**) Asymmetric IgG with different light chains [156]; (**H**) Asymmetric IgG-like molecule with a Fab arm and an scFv arm to eliminate light chain resorting [157,158]; (**I**) Asymmetric IgG using cross-mab technology for LC fidelity and extra Fab arm to make a 2:1 (target cell antigen:CD3ε) antibody call “TCBs,” for “T-cell Bispecifics” [159,160]; (**J**) Chugai’s asymmetric IgG using “Asymmetric Re-engineering Technology–Immunoglobulin” (ART-Ig^®^) platform [161,162] technology, with an scFv fused to one HC to make an ART-Ig^®^-scFv 2:1 (target cell antigen:CDε) antibody; (**K**) tetravalent, bispecific tandem diabody (TandAb) [163]; (**L**) tetravalent, bispecific ADAPTIR™ platform with two different scFvs fused to each Fc [164].

Fundamentally, there are two major types of bispecific antibodies used as TRBAs, bispecific antibody fragments (e.g., Figure 7A–D) and IgG-like asymmetric heterobispecific antibodies (e.g., Figure 7E–H). There are many variations on these two themes, some of which are absolutely critical to the unique function of the antibodies. A sampling of these platforms is shown in Figure 7. Additional details on these various platforms can be found in various recent reviews [60,61,62,149,150,151,152]. This section will describe a few of these platforms briefly and how structure can be very important to the function of TRBAs.

### 3.2. Bispecific Bivalent Antibody Fragments Used to Make TRBAs

Most of the bispecific antibody fragments in development today are based in some manner on scFv antibodies, discovered independently by two research groups in 1988 [46,47], which are comprised of a V_H_ domain linked to a V_L_ domain via a short, flexible linker. It is notable that Huston et al. [47] utilized the (GGGGS)_3_ flexible linker to fuse V_H_ to V_L_; this “gly-ser” linker or variations thereof, is one of the most widely used linkers in bispecific antibodies today. From both an historical perspective as well as a therapeutic perspective, the most significant types of bispecific antibody fragments are tandem scFvs [165], bispecific T cell engagers (BiTE^®^s [166]), dual affinity retargeting (DART^®^s [153]) antibodies, diabodies [44] and tandem domain antibodies [167]. Additional recently described fragment-based bispecific antibody constructs, such as HSA-antibody fragment fusions [168], bispecific killer engagers (BiKEs) [169,170,171], trispecific killer engagers (TriKEs) [169,170,171,172] and dock-and-lock Fabs [173] have also been developed as bispecific antibody fragment platforms. There are several recent reviews that describe these bispecific antibody fragment platforms in detail [60,61,62,149,152,174].

As previously mentioned, the anti-CD19 × anti-CD3 BiTE^®^, blinatumomab, has been approved for commercial use under the trade name of Blincyto^®^. Including blinatumomab, there are currently 27 bivalent, bispecific antibody fragments being tested as TRBAs in clinical trials. Of these, 11 are short-half-life BiTE^®^s or bispecific scFv-based molecules similar to BiTE^®^s, six are next-generation half-life extended BiTE^®^s (i.e., BiTE^®^-Fcs), one is a short half-life DART^®^ construct, three are long half-life DART^®^-Fc molecules, three are immune-mobilizing monoclonal TCRs against cancer (ImmTACs) [155], one is a trispecific killer engager (TriKE) [147] and two are a Trispecific T cell Activating Constructs (TriTACs) [175].

### 3.3. Bispecific Bivalent Asymmetric IgG-Like Antibodies Used to Make TRBAs

One of the most widely utilized approaches for making bispecific antibodies today to be used as TRBAs is the generation of asymmetric heterobispecific IgGs containing two different types of heavy chains. Two important components are required to make asymmetric IgG-like bispecific antibodies that can be developed and manufactured in a consistent manner. The first is the preferred formation and/or isolation of the asymmetric heterodimerized heavy chains (HCs) over the parental IgG homodimeric antibodies [60]. The second is the proper pairing of the light chains (LCs) of each arm with the cognate HC [176].

#### 3.3.1. Asymmetric Pairing of HCs

It is well established that the primary driver for HC dimerization is the high affinity (ca. 10 pM [177,178]) interaction between the C_H_3-C_H_3 domains [60,156,177,178]. The interactions between the C_H_3-C_H_3 domains, which bury over 2400Å^2^ of surface area [178], are driven by a strong central hydrophobic core surrounded by a series of charged residues that provide electrostatic interactions between the two Fc domains [60]. Essentially, three basic strategies have been used to promote asymmetric heterodimerization over formation of the parental homodimers: (i) the first based on physical/spatial interactions, for example, adding a protrusion to one heavy chain and a corresponding cleft in the other (e.g., “knobs-into-holes”) [52,53,54]; (ii) the second depends on alteration of specific amino acid interactions at the C_H_3-C_H_3 interface [60,156,179] and (iii) the third focuses on charge, that is, changing charged amino acid residues to generate a repulsion of homodimers and a corresponding charge attraction between the heterodimer pairs [178,180,181].

Several different platforms have been used to make asymmetric heterobispecific IgG-like antibodies to be used as TRBAs. The first was the three-way fusion of a mouse B-cell, a rat B-cell and a myeloma cell to form a quadroma cell line (Triomab^®^ technology) [53] (Figure 7E). In this platform, the LCs naturally sort to the proper heavy chains due to species specificity. The downside, of course, is that any antibody made using this approach will be highly immunogenic, as mentioned previously was one of the problems with catumaxomab. This platform also was used to generate the first asymmetric heterodimeric IgG-like TRBA to be clinically tested in 1995 [51].

All of the rest of the asymmetric bispecific IgG-like platforms depend on engineering of the Fc to promote either formation of or purification of, the heterodimeric IgG over the parental homodimeric IgGs. The first *bona fide* bispecific IgG-like antibody platform was the knobs-into holes (KIH) platform [52,53,54]. Other platforms, which rely on charge attraction/repulsion, include the electrostatic steering (ES) platform [178], ES plus hinge mutations [180], the Oncomed IgG2-based ES “Bimab” technology [182] and the Chugai “Asymmetric Re-engineering Technology—Immunoglobulin” (Art-Ig^®^) platform [161,162]. Other asymmetric heterobispecific IgG approaches include the modification of specific amino acid pairings in the interface of the C_H_3 domains, such as the Duobody^®^ approach [156], the Zymeworks azymetric platform based largely on hydrophobic interactions [179], the BEAT (bispecific engagement by antibodies based on the T cell receptor) platform from Glenmark [157], the Xencor H/A platform [158,183] and the Merus Biclonics^®^ Platform [184]. Additional platforms used to make asymmetric heterobispecific IgG-like antibodies include Regeneron’s modified protein A-binding platform [185,186], the “strand exchange engineered domain” (SEED), which consists of alternating sequences derived from IgG and IgA C_H_3 domains resulting in asymmetric but complementary pairs, AG and GA, in a manner that only the heterodimeric protein would bind and fold into an active Fc [187,188] and NovImmune’s kλ-antibody platform, which incorporates common HCs paired with a lambda LC in one Fab arm and a kappa LC in the other Fab arm [189]. In this case, essentially all of the binding activity rests with the LCs [189], exactly opposite of what would be the case using heterologous HCs with a common LC. While there are several additional examples of platforms recently developed to generate asymmetric heterobispecific IgG-like antibodies for example, References [19,20,60,61,62,152,190,191], these will not be further described here.

#### 3.3.2. LC Issue for Asymmetric Heterobispecific IgG-Like Antibodies

Whether by generating hybrid-hybridomas by the fusion of two different hybridomas or by genetic engineering, the introduction into a cell line of four antibody genes encoding two different heavy chains (HCs) and two different light chains (LCs), will result in the formation of ten different potential combinations, only one of which is the desired bispecific antibody, due to promiscuous heavy chain (HC)- light chain (LC) pairing [192]. This LC pairing problem can be reduced to four possible pairings if there is a forced pairing of the two different heavy chains to make an asymmetric, heterologous Fc [59]. Thus, even with the high efficiency formation of the heterodimeric Fc via generation of asymmetric Fcs, there still needs to be a solution for the light chain independent distribution issue.

Multiple solutions have been found to alleviate the light chain pairing issue in asymmetric bispecific IgG-like antibodies. One approach, which is essentially an “avoidance” strategy, is the formation of asymmetric IgG-like antibodies with a Fab arm on one side and an scFv on the other half (Figure 7H), such as Xencor’s Xmab H/A platform [158,183] or Glenmark’s BEAT platform [157]. A second solution is the “common LC” approach, in which both Fabs of the asymmetric IgG-like bispecific antibody possess identical LCs [59,161,184,185,193,194]. Another strategy is to generate differences in the HC-LC interactions by switching out C_H_ and C_L_ domains on one half of the antibody to generate a “CrossMab” (CM) [195,196,197]. The result of this switch is the pairing of one normal heavy and light chain on one side of the bispecific antibody and a pairing of V_H_-C_L_-hinge-C_H_2-C_H_3 with V_L_-C_H_1 on the other half (CM^CH1-CL^; [195]). A similar strategy to CrossMab would be to mutate certain sequences in the interfaces of the HC and LC in one and/or the other Fab arm to ensure proper pairing [198,199,200,201].

A final method to control LC distribution is via separate upstream production of the two parental antibodies with post-Protein A recombination of the two antibody halves, typically by reduction and re-oxidation processes [156,180,187], leading to the asymmetric heterodimeric bispecific antibody. Two platforms are built around this concept, the Duobody^®^ platform [156] and the SEEDbody platform [187]. These methods depend on the fact that the heavy chains can be separated via reduction of the interchain disulfide bonds while the HC-LC interactions and disulfide bonds remain stable [156,187]. A variation on this theme is the production of bispecific antibodies in two cocultured strains of *Escherichia coli*, each strain containing a half antibody (HC+LC). After growth of the strains and production of the antibody halves, the antibodies were reduced and re-oxidized to form the heterodimeric IgGs [202]. A mammalian coculture protocol for generating asymmetric heterodimeric bispecific antibodies also has been devised [200], combining aspects of the Duobody^®^ [156] and the bacterial [202] approaches.

Excluding catumaxomab, which had been approved for malignant ascites in Europe but now discontinued, there are currently 21 known asymmetric, bivalent heterobispecific TRBAs in clinical trials that utilize the designs mentioned above, all of which are designed to redirect T cells, via CD33ε binding, to kill targeted cells (Table 2).

#### 3.3.3. Trivalent, Bispecific Antibody Platforms

There are several platforms that have been developed recently to provide two binding arms for target cells and a single binding arm for CD3ε, resulting in trivalent but bispecific, antibodies. The concept behind these antibody formats is to provide better binding to the target cell through avidity, while only providing a single binding arm for CD3ε [159,160], because it is known that providing two binding arms for CD3ε may result in non-specific and undesired T-cell activation [203].

One trivalent, bispecific antibody format is an asymmetric heterodimeric IgG, constructed using the KIH technology, with a single anti-CD3ε Fab arm appended to one of the HCs as described earlier (Figure 7I). LC fidelity is maintained through use of CrossMab technology [159,160]. These 2:1 (target cell antigen:CD3ε) TRBAs have been dubbed by Roche as “TCBs,” for “T-cell Bispecifics.” There are currently two clinical stage TCBs that incorporate the Fab as an extra appendage, including cibisatamab (aka RG7802, RO6958688, CEA TCB) [159,204], which has two binding arms for CEA and a single binding arm for CD3ε and RG6026 (aka RO7082859) [205], which has two binding arms for CD20 and one for CD3ε [160]. Two additional TCBs, one targeting BCMA [206] and another targeting a carboxyl-terminal fragment of HER2 expressed in about half of HER2-positive tumors [207], have been reported but are not yet in clinical trials.

Another trivalent, bispecific antibody is ERY974, which is a silenced IgG1 asymmetric mAb with an anti-CD3 scFv fused to the C-terminal sequence of one of the heavy chains (Figure 7J) [162]. ERY974, which has two binding sites for glypican-3 to optimize avidity but only a single anti-CD3 arm to reduce the chance of non-specific T cell activation [162], is in Phase I clinical trials [208].

Other trivalent, bispecific antibody platforms providing two binding sites for target cells and a single binding site for T-cell CD3ε are “Asymmetric Tandem Trimerbody for T cell Activation and Cancer Killing” (ATTACK) [209] and the new trivalent, IgG-shaped tri-Fab format [210]. These platforms are not yet represented in the clinic.

#### 3.3.4. Tetravalent Bispecific Antibody Platforms

It is generally considered that bivalent targeting of CD3ε can lead to non-specific T cell activation and release of cytokines [203,211], which is not desirable in a T cell redirection platform. Thus, as described in the previous sections, the vast majority of both fragment and IgG-like TRBAs are bivalent, with one antigen combining site binding to the target cell and the other antigen combining site binding to CD3ε on T cells. Having stated that, however, a few clinical-stage platforms stand out as antitheses to that trend. The first of these is the Affimed tandem diabody (TandAb) platform, which is a bispecific, tetravalent tandem diabody with a molecular weight of approximately 114 kDa [163]. Clinical stage TandAbs include AMV564, Aphivena’s anti-CD33 × CD3 TRBA for myelodysplastic syndromes [212] and AFM13, Affimed’s NK-cell redirected anti-CD30 × CD16a candidate for Hodgkin’s lymphoma [148].

The Aptevo ADAPTIR^TM^ platform is a tetravalent, bispecific antibody consisting of two identical scFvs binding to target A fused to the hinges of an Fc and two identical scFvs binding to target B fused via a short linker to the C-terminal sequences of that Fc (see Figure 7L) [164]. The anti-prostate specific membrane antigen (PSMA) × anti-CD3 tetravalent, bispecific ADAPTIR^TM^ TRBA molecule MOR209 (also called ES414) [164] is currently being tested in Phase 1 clinical trials [213] for the potential treatment of prostate cancer. In preclinical studies, even though it possesses two CD3ε combining regions, MOR209 did not appear to activate T cells indiscriminately and it induced lower levels of pro-inflammatory cytokines than some other platforms [164]. Thus, TRBA geometry may play a significant role in whether binding to two CD3ε s on the T-cell surface causes non-specific T-cell activation or not.

Other tetravalent, bispecific platforms not currently represented in clinical trials include IgG-scFv fusions [214], dual variable domain-immunoglobulins (DVD-Ig) [215,216] and Fabs-in-tandem immunoglobulins (FIT-Ig) [217]. IgG-scFv fusions, which are IgGs with scFvs fused to either the C- or N-termini of each HC or LC [214,218,219,220], often suffer from instability due to the unfolding and aggregation of the scFvs, requiring additional modifications to achieve stable, manufacturable candidates [219,220]. DVD-Igs are tetravalent, bispecific antibodies of about 200 kDa comprised of an IgG to which an extra Fv is appended to the N-terminus [215,216]. The Abbvie team that developed the DVD-Ig, however, has moved to the half-DVD platform for their TRBA constructs to reduce non-specific T-cell activation [203]. Finally, the Epimab Biotherapeutics “Fabs-in-tandem immunoglobulins” (FIT-Ig) platform is similar in some respects to the DVD-Ig, except that an entire Fab is appended to the N-termini of each HC [217]. There are no TRBAs in the clinic currently from any of these platforms.

### 3.4. Factors Affecting TRBA Potency

Factors affecting the potency of TRBAs include location of the epitope on the target antigen, size of target antigen, affinity of the TRBA arms to target antigen and CD3ε, valency of the TRBA on the target antigen, antibody size and geometry, antigen density on the target cell [15,17,221], effector-to-target ratios and TRBA concentration [222]. Jiang et al. [222] used a variety of parameters to build a model of the target cell-biologic-effector cell complex (TBE complex) to demonstrate the sensitivities to killing potency with a variety of parameters. One key result that emerged was that TRBA concentration appears to be critical, with a TRBA concentration greater than the K_D_ of the lower affinity binding arm resulting in decreased TBE complex formation due to a shift toward monovalent binding [222]. On the other hand, too high a concentration of TRBAs can result in separate coating of both antigen-positive cells and CD3ε -positive T cells, resulting in poorer killing [223].

While the hierarchy of factors affecting TRBA potency is not entirely understood [17,222], a few factors have become clear over the past several years. First, the epitope for antibody binding to the target antigen is critical, with the best epitopes being membrane proximal [15,17,221,224], especially with larger target antigens [17]. It was demonstrated that targeting a membrane proximal epitope also causes exclusion of the negative regulatory protein CD45 in the synapse, increasing the potency of the T-cell response and killing [15]. This observation that membrane proximal epitopes on the target antigen should provide the greatest TRBA potency has been supported with TRBA-based studies on the cancer targets P-cadherin [225] and ROR1 [226]. Second, the size of the antigen, which can effectively increase the distance within the synapse between the T-cell and target cell, also can affect potency, with much larger targets resulting in lower TRBA potencies [15,221].

It has been shown multiple times that increase in affinity of a TRBA to the target antigen can significantly increase potency [222,225]. Another approach to increase binding of a TRBA to target cells is to increase the avidity, that is, to have more binding arms on the target cell. As described in Section 3.3.3., there are two TRBA formats that provide two binding arms for the target cell with only a single binding arm for CD3ε, the three-Fab TCB format designed by Roche scientists [159,160] and the ART-Ig^®^-scFv format designed by Chugai scientists [162]. The key to understanding whether a 2:1 construct shows better potency than a 1:1 construct is to have each type of construct made for the same target. Bacac et al. [160] compared their 2:1 anti-CD20 × CD3ε versus a 1:1 construct made similarly and demonstrated that the 2:1 format had a 10–100× greater potency in vitro than a similar 1:1 construct. Additionally, the 2:1 format outperformed the 1:1 format in ex vivo assays and showed very potent activity in in vivo animal models [160]. Moreover, Bacac et al. [227] demonstrated that pretreatment with the anti-CD20 mAb, obinutuzumab, prior to treatment with the anti-CD20 × CD3ε 2:1 TCB, RG6026, resulted in significantly lower cytokine release, which may translate into a clinical benefit.

Considering that the anti-CD20 × CD3ε 2:1 TCB format appears to be more potent than various 1:1 anti-CD20 × CD3ε formats [160], it might be interesting to see how other multiple-tumor-target-binding × single CD3ε binding formats might behave. To that end, IGM Biosciences has an anti-CD20 × CD3ε IgM pentameric antibody in preclinical studies that has 10 binding arms for CD20 and one for CD3ε [228]. It will be interesting to follow this highly avid antibody (on the target side) to see how it ultimately compares with the 1:1 and 2:1 formats.

**Figure 8:**
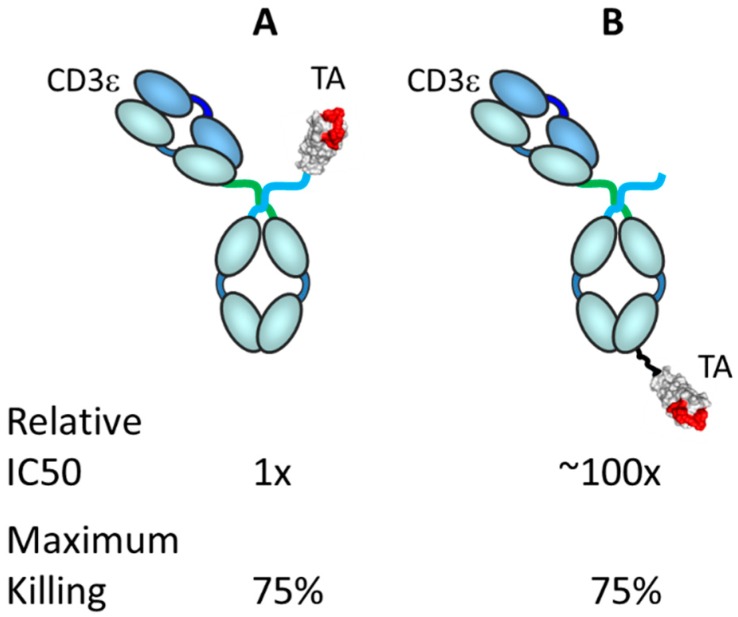
Effect of geometry on in vitro activity of an example TRBA. Cartoon of two antibody-centyrin fusions targeting CD3ε with the Fab arm and a solid tumor antigen with the centyrin (~12 kDa engineered FN3 domain). (**A**). Centyrin fused to the hinge, making it close to the anti-CD3 Fab arm; (**B**). Centyrin fused to the C-terminus of the heavy chain, making it distal from the Fab arm. All components, molecule sizes and in vitro killing assay conditions (E:T 5:1, 24 h assay) were identical. Thus, only the architecture and geometry of the two TRBAs are different, resulting in a ca. 100-fold difference in potency. This simple example demonstrates how important the geometry of the binding arms can be in the design of future TRBAs. Data presented were derived from experiments provided Steve Jacobs, Janssen R&D. These data were previously presented at PEGS Boston, April 2018 with permission.

Significantly, antibody format and size, from small antibodies such as BiTE^®^s (ca. 24 kDa) to much larger formats such as the asymmetric IgGs (ca. 150 kDa), appear to have a lower differential effect on potency than the distance of the epitope to the membrane or affinity to the target antigen [17]. Additionally, binding geometry, which would include target epitope, antibody size and format, binding angle and perhaps other local factors, can influence the potency of the TRBA [17]. In an unpublished study carried out at Janssen R&D, several versions of a bispecific antibody (CD3ε arm)/centyrin (tumor antigen arm) combination were made and tested in vitro for tumor cell killing activity. As shown in Figure 8, the position of the tumor antigen binding arm on the molecule, that is, the geometry/distance of binding between the CD3ε arm and the tumor antigen arm, made a ca. 100-fold difference in in vitro killing activity.

Another area that has not yet been fully investigated with respect to T cell redirection is the role of Fc functionality. The Triomab^®^ platform on which Removab^®^ was designed has a highly active Fc domain that interacts with human FcγRs to increase the immune response [229,230]. It is generally accepted that the presence of an active Fc in a T cell redirecting bispecific antibody would increase the likelihood of pro-inflammatory cytokine release by T cells and other effector cells in the tumor microenvironment [229,230]. The release of these pro-inflammatory cytokines is thought to be part of the therapeutic mechanism of action of these antibodies [231], so while it is desired, it also needs to be controlled [232]. On the other hand, most of the current fragment-Fc, asymmetric IgG or appended IgG platforms have used muted or silenced Fcs so as not to over-stimulate the immune system via interactions with myeloid effector cells. Even with the absence of Fc activity, many treatments with T cell redirecting bispecific antibodies are accompanied by a cytokine release syndrome (CRS) that needs to be addressed as part of the therapeutic paradigm [232]. Thus, it seems likely that many, if not most, T cell redirecting antibodies made now and in the future, will likely continue to avoid Fc activity to limit the potential for immune-mediated toxicities.

## 4. Ex Vivo T-Cell–Bispecific Antibody Approaches

### 4.1. T Cells Armed Ex Vivo with Bispecific Antibody Conjugates

The tetravalent bispecific TRBAs described in Section 3.3.4. are not the only tetravalent platforms being tested. Based on protein conjugation approaches of Nisonoff and Rivers [233] and the ability to generate monoclonal antibodies via hybridoma technologies [31], several groups in the 1980s–1990s generated heterobispecific antibodies using conjugation methodologies [40,234,235]. The first clinical trial ever run with a TRBA was with an anti-CD3 mAb chemically conjugated to an anti-glioma antigen antibody [49] (Figure 2). More recently, chemical conjugation methods have been used to generate bispecific antibodies from existing antibodies for clinical studies. One method that has been widely used is to react the anti-CD3 antibody, OKT3, with Traut’s reagent (2-iminothiolane HCl) and to treat the second antibody targeting cancer cells with sulfosuccinimidyl 4-(*N*-maleimidomethyl) cyclohexane-1-carboxylate (sulfo-SMCC) [236]. Mixed together at equimolar concentrations, these form 1:1 heterobispecific conjugates between the anti-CD3ε mAb, OKT3 and the targeting IgGs [236,237,238] (see Figure 5F). Based on pioneering efforts by Lawrence Lum and his colleagues at the Karmanos Cancer Institute (KCI), these and similar methods have been used to generate conjugated bispecific IgGs of several anti-tumor antibodies. These include clinical candidates such as anti-HER2 trastuzumab × OKT3 (Her2bi) [239] (clinical trial NCT03406858 [240]), anti-EGFR cetuximab × OKT3 (EGFR-bi) [237] (clinical trial NCT02620865 [241]), anti-GD2 3F8 × OKT3 (GD2bi) [238] (clinical trial NCT02173093 [242]) and anti-CD20 rituximab × OKT3 (called CD20bi) [243]. Note that all of these approaches utilize long-existing antibodies (e.g., trastuzumab, rituximab and cetuximab, antibody 3F8) which are chemically conjugated with the “original” anti-CD3ε antibody, OKT3 [244].

All four of these heterobispecific conjugates have been, or are currently being, evaluated in clinical trials by mixing them ex vivo with patient-derived leukapheresed T cells to “arm” and activate those T cells, followed by the administration of the armed and activated T cells autologously to the patients from which they were isolated [21]. Other than various academic efforts, very little has been done to advance chemically coupled bispecific antibodies. Newer technologies, however, may improve the coupling procedures to allow for greater efficiency and the potential for manufacturability. One such method recently described to generated chemically coupled bispecific antibodies with high efficiency was the use of sortase enzyme, which recognized the LPxTG peptide sequence, in conjunction with click chemistry [245]. This approach allowed for the high efficiency formation of a bispecific antibody comprised of two fully active, heterologous anti-influenza IgGs using the mild conditions of an aqueous environment at room temperature [245].

### 4.2. Cytokine-Induced Killer Cells

Another ex vivo approach combines the power of a subset of activated killer cells with the redirection provided by a bispecific antibody. In this case, CD3^+^CD56^+^ natural killer (NK) cells, often described as NKT cells [246], are isolated from a patient’s PBMCs (i.e., autologous), activated ex vivo using anti-CD3 Mabs and cytokines, typically IL-1, IL-2 and IFN-γ and then combined with a bispecific antibody with one arm binding the CD3ε on the surface of the cytokine-induced killers (CIKs) and the other arm targeting a cancer cell surface protein (e.g., CD19, CD20, HER2, EGFR and so forth). This preparation is then administered to the patient to redirect the autologous CIKs to the targeted tumor. While several examples of this approach have been tried in clinical trials, currently the most advanced therapy is an anti-MUC1 × anti-CD3ε bispecific antibody that is dosed in conjunction with autologous CIKs at the Fuda Hospital in Guangzhou, China in collaboration with Benhealth Pharmaceutical Co., Ltd. (clinical trial NCT03554395 [247]).

CIKs are a particularly useful cell phenotype for immune-oncology uses. They possess properties of both T-cells and NK cells, the latter of which can kill target cells in an MHC-independent manner and are primed to kill upon target engagement without the need of additional signaling and stimulus [246]. Key molecular drivers of their cytotoxic activities are the NK receptors NKG2D, NKp30 and NKp46 [246]. In addition, their direct cytotoxic killing through cytotoxic granule secretion is accompanied by robust cytokine secretion of IFN-γ, TNF-α and IL-2 that further support the overall activated immune state in the tumor microenvironment [246].

## 5. Other Examples of Immune Cell Redirection

### 5.1. Early Immune Cell Redirection Efforts

As far back as ca. 2000, several preclinical and clinical efforts were made to use bispecific antibody approaches to redirect CD64 (FcγRI)-bearing immune cells (monocytes, macrophages, activated neutrophils) to kill cancer cells [248,249,250]. One of these, MDX-H210, an anti-CD64 × anti-HER2 bispecific antibody, reached Phase 2 clinical trials [248] but ultimately was discontinued. There are currently no CD64-based bispecific antibody redirection programs in clinical trials. Similarly, there were efforts to utilize bispecific antibody approaches by targeting the IgA receptor, CD89, to redirect activated neutrophils to kill cancer cells [251,252,253,254] and HIV [255]. As yet, none of these efforts has yet resulted in a clinical development candidate.

### 5.2. NK Cell Redirection, BiKEs and TriKEs

Immune cell redirection is not necessarily limited to T cells. There have been efforts since the 1990s to redirect other types of immune cells using targets such as NKG2D [256] and CD16a [257,258] or other surface markers on natural killer (NK) immune cells. NK cells are perhaps the most efficient immune cell killing machines available. There is a long history of therapeutic antibodies using NK cell-mediated antibody-dependent cellular cytotoxicity (ADCC) as a major mechanism for killing targeted cancer cells, including rituximab (Rituxan^®^), trastuzumab (Herceptin^®^) and cetuximab (Erbitux^®^), targeting CD20, Her2 and EGFR, respectively [259]. The composition and release of NK lytic granules filled with perforin and granzymes are comparable to those of the cytotoxic T cell subset but without as strong cytokine release [260]. Thus, there has been a recent push to use bispecific antibody technology to redirect CD16a^+^ (FcγRIIIa) NK cells to kill cancer cells [169,170,171,172,261]. One of these molecules, AFM13, an anti-CD30 × anti-CD16a TandAb [262], is currently being studied in Phase 2 clinical trials [263] for the treatment of Hodgkin’s Lymphoma.

One approach that has been revisited recently is to redirect natural killer (NK) cells to the cancer cells using scFv antibodies targeting CD16a (FcγRIIIa) fused via a short linker to another scFv targeting receptors on the cancer cells (e.g., CD19), similar to a BiTE^®^ format. At least a few groups are renaming these bispecific antibodies “BiKEs,” for “bispecific killer cell engager” [170,171]. In some cases, a third scFv against another cancer target (e.g., CD22) is added to increase the targeting ability [173]. These constructs are called “TriKEs,” for “trispecific killer engagers.” Another variation on this theme is to add immune cell-stimulatory cytokines, such as IL-15, in some “TRiKE”–like constructs, resulting in not only broader targeting with the two targeting scFvs but also NK and T cell activation via the activity of the stimulatory cytokine [264]. Other than AFM13 mentioned above, the only other NK cell redirecting antibody approaching clinical trials is the anti-CD33 × CD16a × modified IL-15 TriKE, OXS-3550 (aka GTB-3550; 161533), which is registered with ClinicalTrials.Gov but not yet recruiting [147].

### 5.3. Combining Engineered Cells with mAb Therapy

Recently, a few companies, namely Unum and Nantkwest, have generated recombinant, autologous or allogeneic NKT or T-cells expressing on their surface the high affinity allele (158V) of the CD16a receptor (FcγRIIIa) [25,265] normally expressed on NK cells. These cells are then used in conjunction with existing monoclonal antibody therapeutics that naturally bind FcγRIIIa via their Fc functionality [265]. The signaling domain for these constructs is the natural ITAM found in FcγRIIIa [265], different from most CAR-Ts today, which utilize CD3ζ as the primary signaling domain.

There are currently three of these types of constructs in clinical trials. The first is Nantkwest’s haNK™, which is a CD16a (158V)/IL-2 expressing NK92 cell line (i.e., allogeneic) currently in Phase I clinical trials by itself [266] and, soon, in conjunction with the anti-PD-L1 Mab, avelumab [267] (NCT03853317; not yet recruiting). Unum Therapeutics has two clinical stage candidates, both of which are derived from patient’s T cells (i.e., autologous approach). The first of these is ACTR707 [268], which is an autologous T cell construct expressing the high affinity allele (158V) of CD16a fused with the CD3ζ activation domain, dosed in combination with rituximab to target CD20^+^ B cells, currently in Phase I [269]. The second is ACTR087 [270], an autologous T cell construct expressing the high affinity variant (158V) of CD16a fused with 4-1BB and CD3ζ activation domains. This construct is being dosed with either rituximab [271] or with Seattle Genetics’ anti-BCMA antibody, SEA-BCMA [272], a humanized, non-fucosylated antibody with significantly increased affinity to CD16a over a normal IgG1 [273]. In all of these cases, the antibodies and recombinant cell are administered separately, so there is no need for formulating for combination therapy.

The potential issue with this approach is that the CD16a-positive T-cells will interact with all IgGs in circulation that can naturally interact with CD16a, including serum IgG1 and IgG3 isotypes which, when combined, make up approximately 10 mg/mL of CD16a-interacting “circulation matrix” IgG. A therapeutic IgG1, such as rituximab, typically is present in circulation at concentrations below 100 µg/mL, so it would comprise about 1% or even less, of the total molecules of antibody vying for binding to the CD16a on the surface of the engineered therapeutic T cells. On the other hand, antibodies engineered to possess a 10-fold (or more) higher affinity to CD16a, such as those with either low or no fucose glycans attached at N297 in the C_H_2 domain [273], might be better candidates to be used in conjunction with the CD16a-modified NK/T-cell therapeutics. It is also possible for auto-antibodies present in the matrix of IgGs to interact with the CD16a engineered T cells and drive autoimmunity. Although typically these auto-antibodies would not cause problems as a result of the natural check-point systems in place with NK cell that express CD16a, the engineered T-cells may not be governed by those same checks [274].

### 5.4. Engineered T or NK Cells with Recombinant Target-Specific TCRs

Another approach to targeting tumor or viral antigens is to utilize the natural TCR machinery of αβT cells but with enhancements to increase the ability of the T cell to kill targeted cells. As differentiated from all of the other approaches in this paper, this strategy is both TCR and pMHC-dependent. Also, in this case, the antigens are typically intracellular and are displayed as neoantigen peptides on MHC I [275]. Different from CARs, the engineered TCRs replace the natural αV-βV of the TCR, they signal through the ITAMs of the TCR complex and their target is the pMHC on the surface of the targeted cells (see Figure 1F). The potential upside of this approach is that the regulatory apparatus of the TCR is in place. The downsides, however, include the possible loss of target with MHC loss or down-regulation on cancer cells as noted earlier [6,12], HLA restriction and the requirement for HLA matching and the effects of a repressive tumor environment, including T cell checkpoints [275]. Nevertheless, there are at least 20 clinical candidates in which autologous T cells from patients have been collected, engineered ex vivo with modified TCRs and then re-administered to the patients [275]. Cancer targets for these clinical stage–engineered TCR T cells include: preferentially expressed antigen in melanoma (PRAME), New York esophageal squamous cell carcinoma 1 (NY-ESO), melanoma-associated antigen (MAGE) A4, MAGE A3/A6, MAGE A10 and alpha-fetoprotein (AFP) [276].

The candidates noted above are all autologous. A different approach using engineered TCRs is the introduction of the TCR machinery in the stable NK-92 cell line to produce an allogeneic cell line that could be used as an off-the-shelf NK-TCR product [276,277]. While this approach is very attractive, it is still in the early stages of development.

## 6. Chimeric Antigen Receptor (CAR)-T and NK Cells

### 6.1. Introduction

There are at least 225 unique, documented CAR constructs currently being tested in clinical trials. Over 90% of the current clinical stage CAR-based cell therapies are autologous CAR-T cell products generated from αβ-T cells (Table 3). Of these, 176 are single CAR constructs per cell, dosed alone. Another 30 clinical candidate CARs either have multiple CARs targeting different tumor antigens per cell or are multiple CAR-T cell lines targeting different tumor antigens and mixed for dosing. One clinical stage autologous CAR construct is made using NKT cells. Finally, 14 CAR constructs in clinical trials are allogeneic, 11 of which are from T cells and 3 from NK or NKT cells (Table 3).

As mentioned previously, two CAR-T cell products, Kymriah^®^ (Tisagenlecleucel-T; aka CTL019, CART-19) [104] and Yescarta^®^ (Axicabtagene ciloleucel; aka KTE-C19) [105], have been approved by the US-FDA, both in 2017. Both of the approved CAR-T products target CD19, with Kymriah^®^ approved for treatment of B-cell ALL and, more recently, DLBCL and Yescarta^®^ approved for treatment of DLBCL. The most advanced clinical candidates not yet approved are bluebird bb2121 [278] and JCAR017 [lisocabtagene maraleucel or liso-cel] [279], both in Phase III.

Nearly half (106/225) of the clinical stage CAR-T clinical candidates have originated in China, with the remainder originating in the US (96) or rest-of-the-world (23) (WR Strohl, BiStro Biotech Consulting Antibody and CAR-T Database, last updated 20 June 2019). Interestingly, over half (125/225, ~56%) of current CAR-based clinical trials are sponsored and run by academic groups, government institutes or hospitals without apparent industry funding; the remainder (100/235, ~44%) are either sponsored by biotechnology or pharmaceutical companies or are collaborations between medical or academic institutions and industry (WR Strohl, BiStro Biotech Consulting Antibody and CAR-T Database, last updated 20 June 2019). The fact that over half of the clinical trials are investigator-driven is significant, as it demonstrates the leading role that academic (e.g., University of Pennsylvania), medical/institutional (e.g., MD Anderson Cancer Center, Fred Hutchinson Cancer Research Center, Memorial Sloan Kettering Cancer Center) and government (e.g., National Institutes of Health) investigators have taken in moving CAR-T therapies forward from an idea to a new and powerful mode of cancer therapy.

### 6.2. Autologous CARs

We have briefly described the first, second and third generation of CAR-T cells (Section 1.3; Figure 2 and Figure 3) and there are many reviews of the autologous CAR-T process [22,23,24,280,281,282,283], so we will only summarize the details of a few leading CAR-Ts here. Most of the current clinical stage CAR-based cell therapies are autologous CAR-T cell products (Table 3), that is, personalized products that are derived from a patient, activated (e.g., typically using CD3/CD28 beads), genetically modified and expanded ex vivo and then reinfused back into that same patient [282,283]. Vectors used to transduce the T cells include lentivirus [284], replication-defective gamma retrovirus [285] or transposons such as PiggyBac [286] or Sleeping Beauty [287]. Table 4 compares the salient features of the two approved autologous CAR-Ts targeting CD19 as well as the two Phase III autologous CAR-T therapeutic candidates, JCAR-017 [279]—targeting CD19 for treatment of NHL and bb2121 [278]—targeting BCMA for treatment of multiple myeloma. Key features of these CAR-Ts are the source of the cells (patient PBMCs vs defined CD4/CD8 populations), parameters for ex vivo stimulation, vector used to transduce the T cells with the CAR (lentivirus or retrovirus), hinge and transmembrane domains, costimulatory domains and the common use of CD3ζ signaling domain (Table 4). Where known, production from leukapheresis to re-administration to patient took from 10 to 28 days, depending on the candidate and all of these candidates required lymphodepletion with fludarabine and cyclophosphamide treatment, typically two-to-three days prior to administration of the CAR-T therapeutic.

Although the currently approved products are non-standardized mixtures of CD4 and CD8 T cells, studies are on-going to determine the optimal proportion of each cell type [288]. In addition, since these products are not nearly 100% CAR positive, as gene modification efficiencies vary from vector to vector and T cell pool-to-T-cell pool [280], these CAR-T cell products are typically dosed in terms of a standardized number of CAR^+^ cells (Table 4). One further step in the direction of standardization and product homogeneity is JCAR017 (lisocabtagene maraleucel or liso-cel), which is dosed using a fixed ratio of CD4^+^ and CD8^+^ T cells [279,289].

**Table 4:** Brief comparison of autologous CAR-T constructs.

Property	CAR-T Constructs			
	Kymriah^®^ (Tisagenlecleucel-T; CTL019)	Yescarta^®^ (Axicabtagene Ciloleucel; KTE-C19)	Lisocaptagene Maraleucel (Liso-cel, JCAR-017)	bb2121
Sponsor	Novartis	Gilead (Kite)	Celgene	Celgene/bluebird
KEGG Number #	D11386	D11144	Na	Na
Clinical stage	Approved by USFDA	Approved by USFDA	Phase III (NCT03575351)	Phase III (NCT03651128)
Base cost (US)	$475,000 for B-ALL; $373,000 for R/R DLBCL	$373,000	Na	Na
Indication	B-ALL, R/R DLBCL	R/R DLBCL; PMBCL	R/R DLBCL; CLL	MM
T-cell source	Patient PBMCs; autologous; unspecified	Patient PBMCs; autologous; unspecified	Patient CD4 and CD8 T cells 1:1 ratio; autologous	Patient PBMCs; autologous
Vector	Lentivirus	Retrovirus	Lentivirus	Lentivirus
Antibody	Anti-CD19 mouse scFv FMC63	Anti-CD19 mouse scFv FMC63	Anti-CD19 mouse scFv FMC63	Anti-BCMA
Costimulatory domain	4-1BB	CD28	4-1BB	4-1BB
Signaling domain	CD3ε	CD3ε	CD3ε	CD3ε
Hinge and transmembrane	CD8α	IgG1 Fc	IgG4 Fc spacer; CD28tm	CD8α
Other Markers	Nk	nk	EGFRt	nk
Ex vivo activation	CD3, CD28	CD3, IL-2	nk	CD3, CD28
Lymphodepletion	Yes	yes	yes	yes
Time from leukapheresis to infusion	21–28 days	17 days	nk	10 days
Dose	0.2 to 5 × 10^6^ CAR-positive viable T cells/kg	0.4–2 × 10^6^ anti-CD19 CAR-positive viable T cells/kg	5 × 10^7^ CD8^+^ and 5 × 10^7^ CD4^+^ CAR-positive (not weight based)	50–800 × 10^6^ CAR-positive T cells (not weight based)

Abbreviations: B-ALL, B-cell acute lymphoblastic leukemia; BCMA, B-cell maturation antigen; DLBCL, diffuse large B-cell lymphoma; CAR, chimeric antigen receptor; CD, cluster of differentiation; CLL, chronic lymphocytic leukemia; MM, multiple myeloma; Na, not applicable; nk, not known to these authors; PBMC, peripheral blood mononuclear cells; PMBCL, primary mediastinal large B-cell lymphoma; R/R, relapsed or refractory. #: Number. References used to build this table: [278,279,280,281,282,290,291,292,293,294,295].

### 6.3. Allogeneic CARs

There are at least 14 allogeneic CAR-NK, CAR-NKT or CAR-T cells in clinical trials (Table 3). These are all constructed uniquely and have a wide range of properties based on the host cell (NK, NKT or T cell), the genes knocked out and how the CARs are made. For allogeneic CAR-Ts, either primary cells from a healthy subject or stable cell lines from a source other than the treated patient are used.

These allogeneic cell products will not naturally be histocompatible and may either be rejected by the patient’s immune system or cause graft versus host disease (GVHD) in the case of T cell products. To prevent rejection, the major source of histocompatibility mismatch, the class I HLA surface proteins, can be deleted by genetic engineering [20,296,297]. To prevent GVHD, cells that are naturally devoid of a TCR (e.g., NK cells) or T cells in which the TCR has been deleted by genetic engineering can be used [298]. These cells or cell lines are then further engineered to express a CAR, as with the autologous products described in Section 1.3 and above but can now be given to any patient, representing a truly universal cell therapy. First generation allogeneic cell products were derived from NK cells and lacked the potential to elicit GVHD [299]. However, such products would eventually be rejected by the adaptive immune system (T and B cells), limiting their utility.

**Figure 9:**
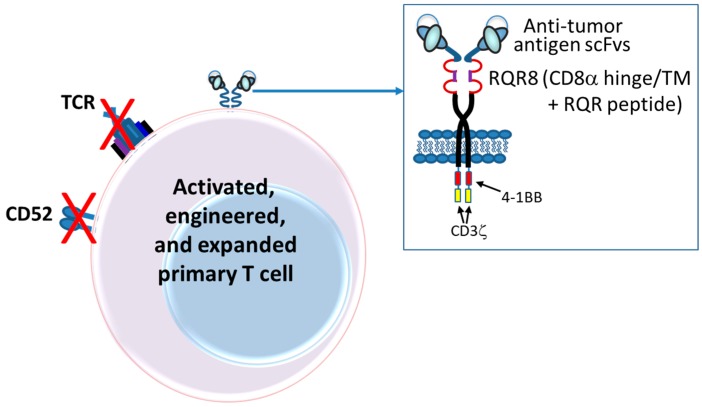
Drawing depicting the construction of an example allogeneic CAR-T cell. The CAR contains, from N-to-C terminus, anti-BCMA scFv, 136 amino acid residue RQR peptide, CD8α hinge and transmembrane (TM) domain, 4-1BB costimulatory domain and CDζ signaling domain in a cell in which TCR- α and CD52 have been knocked out using gene editing technology [301].

The current wave of allogeneic CAR-T cells is perhaps best described as “allogeneic, transiently engrafted T-cell therapeutics.” To give a general sense of how these might be made, the process developed by Cellectis is briefly reviewed here. Allogeneic cells are derived from health donors, are typically engineered to remove the TCR function (for example, by knockout of *TRAC*, eliminating TCR-α) and the pan-lymphocyte maker, CD52 (target of alemtuzumab) but are not made HLA Class I deficient (Figure 9) [300,301]. Prior to treatment, the patients are lymphodepleted using alemtuzumab and then the allogeneic cells, which are resistant to alemtuzumab via CD52 knockout, are dosed. Over time, the patients’ immune systems will naturally be restored and with that, the HLA^+^ allogeneic cells will be eliminated [300]. The manufacturing method for BCMA-targeted, allogeneic CAR-T cells was described as an 18-day process including apheresis from donors, activation, transduction, TALEN gene editing, expansion, removal of TCRα^+^ cells and finally storage [300].

An additional level of safety has been incorporated into the Cellectis allogeneic CAR-T cells. The RQR8 polypeptide, which contains two anti-CD20 rituximab epitopes and a single anti-CD34 Qbend10 epitope [302] has been incorporated into the hinge portion of the CAR, which can be used as a target for rituximab killing of the allogeneic CAR-T (Figure 9) [301,303]. While this first generation of allogeneic CAR-Ts is perhaps not a truly “off-the-shelf” product manufactured from stable cell lines like a biologic drug, it is a significant step in that direction.

Next generation allogeneic products utilizing state-of-the-art genetic engineering technologies in which both the class I HLA and TCRs have been deleted are approaching the clinic. Because these cells will not likely be recognized by the immune system, it will be imperative to include safety switches or even multiple orthogonal kill switches, in such products. It remains to be seen whether these gene edited allogeneic products will be as effective as autologous products and whether they represent a commercially viable source of engineered cell products [304].

### 6.4. Alternative Cell Types for CAR Expression

T-cells are not the only cell type that is being explored for engineered immune cell therapies. Natural killer cells (NK), natural killer T (NKT) cells and even macrophages expressing CARs are all being tested in the clinic [305]. Like T cells, NK and NKT cells share many of the killer functions of T cells but have differences that can be either an advantage or disadvantage, depending on the situation. NK cells are derived from the same immunological precursor cells as T cells and, therefore, have many of the same activities [306,307]. The most obvious difference is that NK cells do not possess a TCR. As a result, it is possible to use NK cells in an allogeneic setting, as the risk of GVHD associated with a mismatched donor TCR does not exist [299]. However, NK cells, unlike T cells, do not robustly expand and do not persist in vivo. In scenarios where the tumor target antigen is expressed at a low level on normal tissues, this could be an advantage, as the product would have a limited life span and penetrance *in vivo*. However, the current dogma is that persistence is important to maintain good responses and long-term remissions, which is not afforded by NK cell-based products [299,306,307]. NKT cells, on the other hand, share properties of both NK cell and T cells. Although NKT cells will not persist like T cells, they do undergo expansion in vivo [308]. Invariant NKT (iNKT) cells—A subclass of NKT cells—Express a TCR that does not recognize classical MHC I presented antigen but, instead, recognizes non-classical MHC I presented antigens that are mainly confined to infectious agents. Therefore, iNKTs are not expected to elicit a GVH response, similar to true NK cells.

Gamma/delta (γ/δ) T cells are very similar in surface marker expression and function to NKT cells in that the γ/δ TCR does not recognize classic MHC I-peptide complexes and are also thought to be useful in an allogeneic setting [309]. There do not appear to be any γ/δ-CAR-T cells currently in clinical trials, although there is one registered clinical trial for collecting γ/δ-CAR-T cells as a feasibility study for constructing CD33-CD28 γ/δ-CAR-T cells [310]. The company involved with that trial, TC Biopharm, also claims to have a non-recombinant allogeneic γ/δ-T cell product in Phase I clinical trials for AML (no NCT provided). Additionally, other companies such as Gammadelta Therapeutics and Gadeta are also working with γ/δ-T cell therapeutics and Lava Therapeutics is making TRBAs that recognize γ/δ-T cells instead of αβ-T cells, so it is expected that TRBA and/or CAR-T products utilizing the unique biology of γ/δ-T cells will soon be tested in clinical studies. CAR constructs employed with NK, NKT, iNKT and γ/δ T-cells utilize the same signaling molecules as αβ TCR T-cell CARs [311].

Macrophages represent the newest cell type being engineered for cell therapies. Unlike lymphocyte-derived cell lineages, monocyte-derived lineage cells like macrophages function mainly as professional antigen presenters and in the removal of invaders through phagocytosis [312]. Engineering macrophages with CARs that signal through a FcγRIIIa (CD16a) receptor intracellular domain imparts on the cell the ability to recognize, phagocytose and then present tumor cell antigens to other T cells to further amplify the immune response. Although not efficient killers, engineered macrophages have the ability to broaden the immune response to the tumor cells. Carisma Therapeutics is on track to reach the clinic with their first CAR-macrophage in early 2020.

A key shortcoming of many of these alternative cell products is the quantity of each cell type present in normal, let alone compromised, patients. The ability to generate a sufficient amount of product for each patient will have to be significantly improved [313].

### 6.5. CAR Designs

#### 6.5.1. scFvs

The first CAR-T cells were nearly universally constructed using mouse-derived scFvs [76,281]. In fact, both of the approved CAR-T constructs, Kymriah^®^ (Tisagenlecleucel-T; aka CTL019, CART-19) [104] and Yescarta^®^ (Axicabtagene ciloleucel; aka KTE-C19) [105], as well as the Phase III candidate, JCAR017 (Table 4) and many of the other 86 CD19-targeted CARs in clinical studies, utilize the same mouse-derived anti-CD19 scFv, FMC63 [281]. One of the first CARs to be made with a humanized scFv was a CAR-T targeting HER2, which utilized an scFv derived from trastuzumab [314]. Only in the past several years have the antibodies for incorporation into CARs been constructed using humanized or human scFvs, a practice that should grow significantly.

The selection and engineering of antibodies for scFvs is actually much more important than many CAR-T engineers have historically perceived. Many of today’s CARs were constructed with antibodies that were retrieved from the freezer, the literature or from academic colleagues. Most of those antibodies were not designed or engineered for stability, lack of aggregation or optimized folding as scFvs or even, in many cases as noted above, for humanization. The reformatting of an IgG antibody into an scFv, although currently reduced to practice, can result in a less stable domain compared to the antibody from which it was derived [315]. As noted earlier, scFvs are notorious for their instability, unfolding, domain swapping and aggregation [220]. The consequence of this loss of stability can have functional and toxic consequences, as a poorly stable binding domains can aggregate and lose binding capability when on the cells surface or cause non-target-specific, tonic signaling [316]. Thus, scFv stability, as well as human-ness, epitope targeting and affinity, are key issues that need to be addressed in the design of future scFv-derived CARs.

#### 6.5.2. Domain Antibodies and Alternative Scaffolds

As with the extensive protein engineering that has helped evolve the TRBAs, creative formatting of CARs and their uses in CAR-T cells has helped expand the capabilities of functional control of CAR T-cell products going into clinical development [317]. Most current CARs utilize scFv-formatted antibody fragments for tumor targeting. Recently, alternatives to scFv formats have been explored, some making it into full clinical development. Humanized llama or camelid V_HH_ fragments or single domain human antibody fragments, that can be engineered to possess all of the binding selectivity and specificity of full V_H_/V_L_ dual-domain antibody fragments, are becoming more frequently used for tumor targeting [318,319]. V_HH_ domains possess the added benefit of simplicity afforded by the single domain, as compared to the two domains separated by a linker of the more typical scFv.

In addition to single domains, others have been pursuing alternative scaffolds based on fibronectin repeats (e.g., Adnectins or Centyrins) [320] and ankyrin repeats (DARPINs) [321,322]. Similar in their simplicity, these alternative scaffolds possess many of the binding properties of antibodies and antibody fragments. Human and humanized versions of these scaffolds have lessened some of the anxiety associated with their use in clinical settings, although with the exception of Centyrins [323], none have been tested in the clinic. The potential to use additional scaffolds, like Centyrins or Anticalins, is only limited to the imagination, as clinical experiences with other protein-based scaffolds are starting to demonstrate their additional potential use in CAR-T programs.

#### 6.5.3. Multiple CAR Designs

In their simplest forms, CARs target only one tumor associated antigen. However, multiple targeting domains can be linked together on one CAR to generate multi-specific CARs. Typically separated by the common G_4_S linker that is used for scFv engineering, “beads-on-a-string” designs linking scFv or single domain binding elements have produced CARs that can be activated to kill target cells that express more than one antigen or bind to different epitopes on the same antigen [324,325]. For example, Janssen R&D and Nanjing Legend Biotech have a dual-BCMA-epitope-binding CAR-T product, LCAR-B38M, in Phase 2 clinical development for treatment of multiple myeloma [326,327]. Perhaps more exciting are multiple CARs on the same cell in which each CAR possesses only one signaling domain. CARs that provide signal 1 through a CD3ζ ITAM are not very effective killers and require a second signal provided by the intracellular domains of 4-1BB or CD28. These signaling domains can be split between two CARs, each with a different binding specificity, producing logic-based CAR T-cells. These so-called “and” (target A and target B) CARs only produce robust target cell killing if both target antigens are engaged on the surface of the target cell [328,329,330]. This strategy and other related logic-based designs, for example, the “but not” CAR (target A but not target B) and the syn/notch CAR, are being explored to address the complex nature of solid tumors and to generate safer products [331,332].

**Figure 10:**
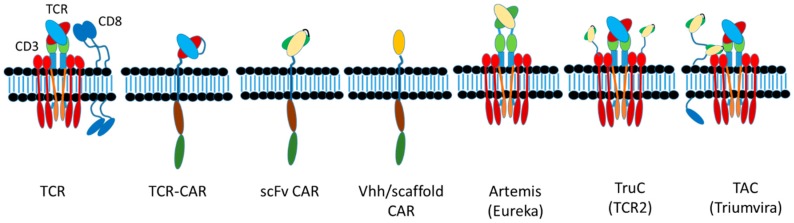
Schematic representation of various CAR formats. Left, natural organization of a CD8^+^ TCR expressed on cytotoxic T cells driving MHC-restricted CD8^+^ T cell effects. TCR-CARs are formed by fusing the TCR α/β variable domains to a second-generation CAR scaffold. The Eureka Therapeutics Artemis platform fuses antibody variable domains to TCR α/β constant regions. TCR2 platform fuses scFvs to CD3ε. Triumvira platform fusing two scFv chains to CD8 stalk, hinge and intracellular domains-one scFv binds to CD3ε engaging the rest of the TCR complex, while the second is free to interact with tumor antigens.

The sophisticated nature of the TCR complex has been shown to be a key mechanism involved in the many biological facets of T cell biology. Attempts to mimic this complex structure using CARs have proven to be difficult [112]. More recently, additional protein engineering designs have attempted to improve on the original CAR concepts. The modular nature of the various Ig-domains that encompass the TCR α/β, CD3δε and CD3γε domains and CD4/8 chains has been open to interesting engineering. The biotechnology company, TCR2, is exploring CD3ε-scFv fusions (Figure 10). Utilizing their TruC platform, the tumor specific binding scFv is fused to the extracellular domain of CD3ε, allowing for complex activation upon scFv-target binding, bypassing TCRα/β-MHC interactions [333]. Similarly, Triumvira has fused two scFvs in place of the MHC I binding head domain of CD8. The more membrane proximal scFv binds specifically to one of the CD3 extracellular domains and the more distal scFv binds to tumor antigens (Figure 10). In Triumvira’s so-called TAC technology, this design essentially locks down the tumor antigen binding to the entire TCR complex structure as a result of the multiple ionic associations of all the players, while simultaneously including the CD8 activities as well [334]. One final spin on this theme is Eureka’s Artemis platform that replaces TCR αV and βV regions with antibody V_H_/V_L_ regions, similar to the very first pre-CAR-like constructs made in 1989 [35]. In this format, antibody specificity is dialed into the TCR complex and can either operate independent of MHC presentation, or, if the antibody variable regions were raised against pMHC, retain the class I restriction, mimicking a true TCR [335]. All of the variations have one primary goal in mind, that is, to more closely resemble the intricate control associated with the natural TCR complex. Future studies will need to be performed to confirm the degree to which these and most certainly others, have achieved that goal.

### 6.6. Additional Enhancements for Tuning CAR-T Cells

In the past few years, CAR-T cells have been made with additional enhancements, either to modulate the immune system, to help the T-cell home to the tumor or to increase the safety of the CAR-T therapeutic [336,337,338]. As noted in Section 1.3, second or third generation CAR-Ts (see Figure 3) to which these enhancements are made are sometimes called “fourth generation CAR-Ts” [338,339,340]. This section describes a few of these “add-ons,” some of which have already become incorporated into clinical candidate CARs.

#### 6.6.1. Safety Switches

As mentioned in Section 6.3, when delivering CAR-T cells that may have potential safety issues, for example, allogeneic CAR-Ts, highly persistent CAR-Ts, CAR-Ts expressing cytokines or CAR-Ts with the capability of generating a strong CRS, it is important for safety reasons to have a method for either killing the CAR-T cells or turning off their function. There have been multiple approaches to this issue, including a variety of different types of kill switches, as well as the application of adapter molecules that link the CAR-T cells to the tumor antigens. Several different types of kill switches have been developed for CAR-T cells, a few of which will be described here. One of the oldest switches is the use of herpes simplex virus thymidine kinase (HSV-TK), which is sensitive to the drug gangcyclovir [341]. Treatment of cells containing HSV-TK with gangcyclovir, however, is quite slow, taking about 72 h to be fully effective and the viral gene product itself can lead to immunogenicity via MHC I presentation, so this approach is not considered to be particularly attractive [342]. A more recent and now more widely used “kill switch” is CaspaCIDE^®^, which couples an inducible caspase-9 (iCasp9) with a small molecule inducer known as AP1903 [98,342,343]. The effect of AP1903 treatment in vivo on cells containing iCasp9 is quick, with 90% loss of iCasp9^+^ cells within 30 min and full 100% effect within 24 h [342]. The iCasp9 kill switch system has been used in CAR-Ts cells since at least 2010 [344] and it is being employed more widely every year since. There are currently at least 11 unique CAR-T cell constructs being tested in clinical trials today that employ the iCasp9/AP1903 system as a safety switch (WR Strohl, BiStro Biotech Consulting Antibody and CAR-T Database, last updated 20 June 2019). A second small molecule kill switch based on caspase-9 is the very recently developed rapamycin-induced caspase-9 dimerization safety switch (iRC9) [345].

Conversely, a different approach to regulate the activity and thus, the safety, of CAR-T cells is to provide the CAR-T system with an inducible “only on” switch, which would rely on the constant presence of a theoretically innocuous small molecule to keep the expression of the CAR gene cassette going. One of these inducible “on” systems is the rimiducid-inducible MyD88/CD24-CAR (iMC-CAR) system [345], which give “on demand” rimiducid-dependent co-stimulation, which enhances proliferation and activation. The CAR, however, is not fully activated until binding of the targeting scFv to the tumor cell surface antigen triggers CD3ε signaling as well [345]. Similarly, there are multiple versions of a tetracycline (doxycycline)-inducible (tet-inducible) CAR, in which CAR expression is completely dependent on the presence of the tetracycline [346,347]. The tet-inducible system, however, appears to be leaky with a significant background expression level in the absence of the inducer [347].

Another effective and popular approach to kill CAR-T cells, when required, is the use of commercially available, approved antibodies targeting tumor targets such as EGFR and CD20. As mentioned in Section 6.3 (see also Figure 9), the RQR8 epitope was constructed as a target for the anti-CD20 mAb rituximab [302] and has been employed in allogeneic cells as a cell-surface kill switch [301,303]. Additionally, a truncated version of epidermal growth factor receptor (EGFRt), which encompasses residues 334–668 of mature EGFR (domains III and IV), has been fused to the GM-CSF leader peptide to make a surface expressed “tag” for CAR-T cells [348,349,350]. This EGFRt tag is recognized by the approved drug cetuximab (Erbitux^®^), which can then be used as a kill switch to eliminate cells expressing the tag [348,349,350]. There are currently at least 12 unique CAR-T cell constructs in clinical trials that have incorporated the EGFRt tag (WR Strohl, BiStro Biotech Consulting Antibody and CAR-T Database, last updated 20 June 2019). This tag can be used not only as a kill switch but also as a marker for sorting and tracking EGFRt-positive cells [348,349].

#### 6.6.2. Adapters

A completely orthogonal way to control the activity of CAR-T cells is to have CAR-T cells that recognize a molecule which is fused to the targeting antibody, that is, an “adapter molecule,” rather than to the targeted antigen itself. In this manner, CAR-T activity only occurs when the CAR-T, the adapter and the targeted antigen are present together [351,352]. Many adapter systems have been described in the literature, including those that use FITC, GCN4 or biotin as the adapter molecule. In these cases, the adapter is conjugated or fused to a tumor antigen-binding scFv (or other binding moiety) and the CAR recognizes the adapter molecule. In this manner, the activity of the CAR-T is controlled by availability of the adapter molecule. A potential significant upside for this kind of molecule could be for use in the allogeneic setting. If one could construct a stable CAR^+^ cell line that targets an adapter, then this could potentially be a universal, allogeneic CAR-T that could be made specific for new targets simply by changing out the scFv that is coupled to the adapter [351,352]. A good example of this is the peptide-specific switchable CAR (sCAR), recently developed, which specifically recognizes a 14-amino acid residue peptide derived from yeast transcription factor GCN4 [353]. The peptide was fused to a Fab targeting CD19 to target B cells and then fused to a CD20 Fab to demonstrate that it too would work similarly [353]. This sCAR platform is an example of how a universal adapter CAR-T system might work.

In a sense, the NK and T cells expressing CD16a [25,265,268,270], described in Section 5.3, would be similar to universal adapter CARs because the only change needed to make a new “drug” would be a different, already approved (or even one in development), targeting antibody [351].

#### 6.6.3. Homing Receptors

At least three critical factors are required to construct a CAR-T cell to treat solid tumors. The first is having the proper tumor antigen target, the second is efficient homing of the CAR-T cells to the tumors and then penetration of the targeting killer cells into the tumors and, finally, the third is a successful defense against the immunosuppressive environment within the tumor microenvironments [354]. These next two sections will cover the latter two of these requirements.

T cells traffic to different tissues based on a wide variety of different homing signals [337,355]. For example, the receptor/ligand pair CXCR4/CXCL12 will help to target T cells to the gut, lung and bone marrow, whereas CCR4/CCL17 will help to home T cells to skin, lung and heart [355]. It is the combination of the proper tissue-based signals and their cognate homing receptors that help home T cells to the proper tissues under the appropriate conditions. While this is a simplified view of very complex biology that requires cell-cell interactions, chemokine gradients and immune signal-mediated upregulation of chemokine receptors, it nevertheless illustrates the importance of specific chemokines and chemokine receptors for homing T cells to target tissues. Most autologous CAR-T cell products currently suffer from the fact that they are very heterogenous, are derived from peripheral blood rather than tumor tissues and are deficient in many or all of the homing receptors required to traffic them to cancerous tissues. To improve the ability of T cells to home to and then penetrate, solid tumors, the proper receptors need to be present; if they are naturally lacking, then they will need to be engineered into the cells.

There are now several examples of attempts to clone chemokine receptors into T cells to improve the ability of those cells to traffic to and penetrate, solid tumor tissues. One of the keys to this approach is understanding which tumors overexpress which chemokines, so that the proper match can be made for each tumor. Chheda et al. [356] demonstrated that the knockout of CXCR3 and leukotriene B4 receptor (BLT1) in CTLs abrogated their ability to home to tumors in mice, which correlated with a loss of efficacy, demonstrating the criticality of those two chemoattractant receptors in CTL migration to the tumors. In a separate study, cultured NK cells were found to have lost the ability to express CXCR2, which led to a defect in trafficking to RCC tumors [357]. When enforced CXCR2 expression was reinstalled into those cells via genetic manipulation, trafficking to the RCC tumors as restored, with a concomitant improvement in tumor cell killing [357].

In an early attempt to manipulate CAR-T cells to home to tumors, Moon et al. [100] transduced mesothelin-targeting CAR-Ts with CCR2, which resulted in more than a 12-fold increase in trafficking of those CAR-T cells to the mesothelin-expressing tumors in a mouse model [100]. Similarly, Siddiqui et al. [358] demonstrated that transduction of T cells with CXCR1 significantly improved the homing of those T cells to tumors expressing CX3CL1 in mice, with concomitant improvement in tumor suppression. In a separate study, both mouse and human pancreatic cancers were demonstrated to over-express the chemokine, CCL22 [359]. Transduction of T cells with CCR4, the receptor for CCL22, led to improved interaction of the T cells with dendritic cells (DCs), increased T cell activation and improved T cell tumor penetration in a mouse model [359], suggesting that addition of this chemokine receptor to CAR-T cells intended for treatment of pancreatic cancer might be fruitful.

Perhaps one of the most critical findings was the apparent requirement for the expression of CXCR3 for extravasation of T cells from the vasculature into the tumor in a mouse model [360]. Neither CCR2 nor CCR5 could fulfil that role, indicating the likely requirement for transduction of multiple chemokine receptors into CAR-Ts intended to treat solid tumors, each with its own function along the path from circulation to tumor tissue. Additionally, these studies exemplify the need to understand which chemokine attractants are expressed in which tumors under what conditions. This information will help to design and construct “smart” CAR-Ts for targeting solid tumors.

#### 6.6.4. Counteracting PD-1/PD-L1-Based Immunosuppression

Not only are trafficking to the site of a tumor and tumor penetration critical to the success of CAR-Ts for solid tumors but also dealing with the typically strong immunosuppressive tumor microenvironment (TME) [111,337,338]. The immune-repressive environments especially found in the TME of solid tumors is clearly a potential issue for causing immune suppression of both CAR-T cells and TRBAs. It is known that PD-1 interaction with PD-L1 has the ability to override the activating signals from CD28, providing a dominant immunosuppressive effect in the TME [361]. To this point, it has also been demonstrated that anti-PD-1 antibodies can rescue the CD28 function in animal models [138]. Thus, exogenous anti-checkpoint target antibodies are being tested in clinical trials to counteract the suppressive immune environment for either TRBA therapy [139,140,141] or CAR-T therapy [142].

With CAR-T constructs, there are additional opportunities to address the PD-1/PD-L1-based immunosuppression by modification of the CAR-T cell itself. There are multiple approaches to this concept. First, there are several reports of CAR-Ts in which either PD-1 or PD-L1 were knocked out [146,362,363,364,365] or modified to generate of a dominant-negative PD-1 lacking the signaling domain [138]. In a slightly different twist, Liu et al. [366] described the construction of a “PD1CD28” CAR-T cell in which the extracellular domain of PD-1 was fused to the intracellular activating domain of CD28. With these cells, the presence of PD-1 in the TME would act as a stimulatory signal through the CD28 signaling domain [366]. Finally, CAR-T cells have been engineered not only with the CARs but also with the ability to express and secrete into the TME either anti-PD-1 antibodies [144,145], anti-PD-L1 antibodies [143], anti-CTLA4 antibodies [145] or decoys to block PD-1/PD-L1 interaction such as the C_H_3-PD-1 fusion [367].

In a different twist to dealing with the PD-1/PD-L1 immunosuppressive axis, Xie et al. [364] generated CAR-T cells that target PD-L1 in the tumor microenvironment. In this way, they not only were targeting PD-L1-positive cancer cells but also stromal cells in the TME that help provide for the immunosuppressive environment. In targeting PD-L1, Xie et al. [364] found that CAR-T cells themselves produced a low level of PD-L1, so knock-outs of endogenous CAR-T PD-L1 were made that were significantly better than wild-types. The PD-L1 CAR-T cells reduced tumor growth and increased survival in an animal model. A clinical candidate CAR-T cell targeting PD-L1 has been registered but not yet recruiting patients, for the treatment of non-small cell lung cancer (NSCLC) [368].

#### 6.6.5. Cytokine-Expressing CAR-Ts

Another approach to dealing with the immunosuppressive environment of solid tumors is the generation of CAR-T cells that produce T cell stimulatory cytokines themselves [111,338]. A variety of cytokine-expressing and secreting CARs have been made and tested preclinically [340]. These constructs have been given names such as “armored CARs” [340] or TRUCKs (i.e., T cells redirected for universal cytokine killing) [99,339,369]. Koneru et al. [370] reported the generation of CAR-T cells targeting MUC-16^ecto^ which also expressed and secreted IL-12 to the TME, which now has been taken into Phase I clinical trials [370] where it is being delivered directly into the tumor [371].

## 7. Targets for Clinical Stage TRBAs and CAR-T Cells

As shown in Table 1, there are currently 289 unique TRBAs and CARs being tested in clinical trials today, targeting a total of 53 unique targets (BiStro Biotech Database, last updated 20 June 2019). Currently, the 61 unique TRBAs in clinical trials target 31 different antigens, with seven candidates targeting BCMA, six candidates targeting CD33, five candidates each targeting CD20 and CD123 and four candidates targeting PSMA (Table 5). A total of 18 different targets are currently being targeted each by a single known TRBA clinical candidate. Of the 61 clinical stage TRBAs, 31 (~51%) primarily target heme malignancies, 25 (~41%) primarily target non-central nervous system (non-CNS) based solid tumors, four target neurological tumors and one targets human immunodeficiency virus (HIV). There are only two clinical-stage TRBAs currently targeting CD19, blinatumomab, which has been approved under the trade name Blincyto^®^ for treatment of B cell acute lymphoblastic leukemia (B-ALL) and AMG 562, a half-life extended BiTE^®^ construct in Phase I clinical trials [372].

On the other hand, of the 225 clinical candidate CARs, 88 (39%), including the two approved CAR-T therapeutics, primarily target CD19 (of these, 11 target CD19 plus at least one additional B-cell target). Other tumor antigens most frequently targeted by CAR constructs include BCMA (26 candidates targeting), mesothelin (12), GD2 (10), CD123 (8), CD22 (8) and HER2 (6) (Table 5). Approximately 67% of all clinical stage CAR constructs target hematological cancers, largely driven by the vast number of CD19 targeted clinical candidates. Altogether, CAR constructs are being studied in clinical trials against 43 known unique targets.

As mentioned in Section 5.3, there are three clinical candidate recombinant NK or T cells that express Fc**γ**RIIIa to be redirected by therapeutic antibodies to tumors. Two of these candidates target CD20 for NHL and one targets BCMA for MM. As noted previously, a clinical trial of recombinant Fc**γ**RIIIa^+^-NK-92 cells with the anti-PD-L1 mAb, avelumab [267], is already planned, which could potentially extend this approach to solid tumors. If this combined cell/targeting mAb approach yields significant efficacy in the clinic, this number could rise quickly, because the cell products themselves would not need to be changed. The existing cell product candidates would just need to be paired in clinical trials with different approved therapeutic antibodies, such as trastuzumab for HER2-positive tumors, daratumumab for CD38-positive multiple myeloma (this would work for T cells but not for NK-92 cells, which are CD38-positive) and atezolizumab, avelumab or durvalumab for PD-L1-positive tumors and so forth.

An interesting twist on choice of targets is the recent report of using CAR-T cells to target the tumor micro-environment instead of the cancer cells themselves [364]. They generated CAR-T cells targeting the tumor TME-specific fibronectin splice variant, EIIIB [364]. Dosing of the EIIIB CAR-T cells helped to drive immune response to the tumor, suppressing tumor growth [364]. This strategy is not yet in clinical trials but seems promising.

As mentioned above in Section 6.6.3., one of the three critical issues for building successful CAR-Ts is finding the right tumor antigen targets. Due to the potency and toxicity of CAR-Ts and TRBAs to the target cells, it is critical to have either tumor-specific targets or targets that are vastly over-expressed in tumors as compared with normal tissues. The search for truly tumor-specific antigens has been ongoing for decades. Although some antigens have been discovered that are very tumor selective (e.g., MUC1, EGFRvIII, CEA, GD2 ganglioside, PSCA), essentially no antigens have been discovered that are absolutely restricted to tumor cells [373]. As a result, strategies to overcome the potential toxicity associated with killing normal cells that express these targets, even at low levels, are required.

In addition to the paucity of truly tumor-specific antigens, there is significant tumor antigen heterogeneity, that is, not all cells within the tumor will possess the targeted antigen, which makes the antigen selection bar even higher [374,375]. For example, antigen presence on cells within a tumor, for example, HER2 in NSCLC tumors, may be present on only 40% of the tumor cells [375]. Moreover, the copy number of tumor surface antigens can vary significantly from cell-to-cell within a tumor, as well. It is common for many cancers, including lung cancer, renal cell carcinoma, breast cancer, AML and CLL, amongst others, to have significant subclonal populations within the tumor [376]. Several genomic studies have revealed the extreme heterogeneity within tumors and even a wide range of heterogeneity amongst patients with a single type of tumor and heterogeneity amongst different types of tumors [377,378]. A mathematical model based on genomic analysis of tumor heterogeneity has even been developed called “MATH” (mutant-allele tumor heterogeneity) score, which has been used to help identify the extent of heterogeneity in various tumor types [378,379]. The problem with tumor heterogeneity, no matter how carefully measured, is that it generally works against any tumor-targeting approach, including TRBAs and CAR-Ts.

Even efficient killing of antigen-positive cells will only eliminate part of the tumor. Thus, it is important to note that there are examples of both CAR-T cells [380] and BiTE^®^s [381] demonstrating bystander killing of antigen-negative cells that were in direct contact with antigen-positive cells. In the case of BiTE^®^-induced bystander killing, FAS and ICAM-1 were both upregulated on the antigen-negative cells, which helped contribute to the bystander killing process that took place over a matter of hours after initial contact of the BiTE^®^ with the antigen-positive tumor cells [381]. It is known that IFN-**γ** can upregulate Fas (CD95) on the surface of cancer cells [382]. Since both CAR-T cells [380] and TRBA-induced T-cell killers [381] both induce the production of IFN-**γ** as part of their activation and killing process, it is likely that Fas-mediated apoptosis of bystander cells may be more prevalent than shown in just these few studies. Additional studies need to be carried out to determine the extent to which bystander killing can help T-cell redirected strategies to eradicate tumors.

For both reasons mentioned above, that is, the need for greater tumor specificity and tumor antigen heterogeneity, one of the key approaches is to build CAR-T cells that have the ability to target multiple antigens, which appears to be part of a trend in tumor targeting going forward [375]. In our most recent analysis, we count 30 “multiple” targeting CARs out of 225 total unique CARs (Table 3), which is ca. 13% of all CARs being tested in clinical trials today.

Highly engineered CAR-T cell products in the future will not only have the ability to regulate what combinations of targets are engaged to drive an activation response but also to control through signal domain optimization the strength of that activation [329,330,331,332]. In any case, using either TRBAs or CAR-T as treatment options, it will be necessary to determine the presence of tumor antigens before treatment and to score for the change in tumor antigens after treatment [373].

## 8. Cytokine Release Syndrome (CRS) and Its Effect on Treatment

Cytokine release syndrome (CRS) is a significant concern for both CAR-T and TRBA mediated therapies [383]. Virtually every CAR-T and TRBA candidate tested in the clinic thus far has had at least some patients experiencing CRS adverse events. For Blincyto^®^, the only currently approved TRBA on the market, CRS occurs in only about 7–15% of patients depending on indication but in some cases, it was quite severe [384]. More severe cases of CRS can present clinical signs resembling severe inflammatory syndromes such as hemophagocytic lymphohistiocytosis (HLH) or macrophage activation syndrome (MAS) [383].

CRS is a major concern for CAR-T cell products. For example, treatment of 101 patients for refractory aggressive B-cell NHL with axicabtagene ciloleucel (KTE-C19) resulted in grade ≥3 CRS in 13% of the patients, whereas treatment of 51 patients with tisagenlecleucel for relapsed or refractory DLBCL led to grade ≥3 CRS in 26% of the patients [385]. As seen in virtually all CD19 CAR-T trials, the rapid expansion and activation upon CAR-T cell product target engagement results in the expression of toxic cytokines including IL-6, TNF-α and IFN-γ [386]. The release of these cytokines can be lethal, unless appropriately managed in the hospital setting. Current management includes immunosuppressive corticosteroid infusion and/or inclusion of an IL-6R blocking antibody (Actemra^®^) [386]. Most recently, the prominent role of IL-1 produced by monocyte/macrophages during CAR-T therapy has been elucidated as a major driver of CRS; potential treatments with IL-1 blocking antibodies are being tested in the clinic [387]. Very recently, it was demonstrated that GM-CSF stimulation of monocytes/macrophages may play a significant role in driving CRS [388,389]. Deletion of the GM-CSF gene in CAR-T cells eliminated CRS and actually improved CAR-T functionality in mouse models [388,389]. Similarly, GM-CSF function could be managed using an anti-GM-CSF mAb, such as lenzilumab [388,389], or one of the other clinical-stage anti-GM-CSF mAbs (e.g., otilimab, namilumab, TJ003234). These promising results suggest that as we learn more about the mechanisms by which CRS occurs, improved management will quickly follow.

It is important to note that for CAR-T cells, CRS is driven by the expansion and activation of the CAR-T cells and that in the early clinical trials in B-ALL, the CD19 target was highly and widely expressed in the total B cell compartment [383]. This may have resulted in an over-activation paradigm that may not be seen in more selectively targeted CAR-T cell products.

CRS often occurs with a short time window (e.g., 0–72 h) after dosing. Two of the key predictors or correlates, of severe CRS with either TRBA or CAR-T therapy are tumor burden and therapeutic dose [383]. For T-cell engaging bispecific antibodies, many investigators are now using step-up dosing (low dose preceding before regular dose) [390] or, in the case of the CD20-TCB RG6026, pre-dosing with the anti-CD20 mAb, obinutuzumab [227]. These types of dosing protocols appear to help lower the incidence of CRS. For CAR-T cell therapeutics, investigators have tried split-dosing to help manage CRS [391,392].

Neurotoxicity is also a significant AE that can occur with both TRBA and CAR-T therapy. Neurological toxicities occur in about two-thirds of all patients taking Blincyto^®^, typically within the first two weeks of therapy [384]. Pretreatment with dexamethasone or concomitant treatment with corticosteroids seemed to help reduce the neurological adverse events [393]. In clinical trials, neurotoxicity associated with CAR-T therapy (CAR-T-related encephalopathy syndrome or CRES) occurred in about 28% of patients treated with axicabtagene ciloleucel (KTE-019) and in 13% of patients treated with tisagenlecleucel (CTL019) [385].

## 9. Comparison of TRBAs and CAR-Ts Therapeutic Approaches

### 9.1. General Comparison of TRBAs with CAR-T Cells

Table 6 shows the overall comparisons of TRBAs with autologous and allogeneic CAR-T cells. Since there is such diversity amongst both TRBAs (e.g., short vs log half-life; bivalent vs trivalent and tetravalent; geometry) and CAR-Ts (affinity of antibodies used; use of different activation domains; methods for production and activation ex vivo, T cell types included, etc.), some the comparisons are necessarily generalized. Several other recent reviews also have compared many of the salient features of TRBAs and CAR-Ts [27,223,394,395].

Fundamentally, it is generally considered that CAR-T cells are more potent and efficacious than current TRBAs [17,127] but this comparison is largely made on Kymriah^®^ and Yescarta^®^ CAR-T therapies versus the only currently approved TRBA, Blincyto^®^. It may turn out that new TRBA therapeutics in development may leapfrog Blincyto^®^ in efficacy and be much more competitive with the CAR-T therapies while maintaining the virtues of TRBAs. When it comes to cost, availability, convenience, ability to control dosing, ability to re-dose over time and the requirement with CAR-Ts for lymphodepletion, however, TRBAs have a significant “usefulness” advantage over CAR-T cells.

From a competitive marketing stance, Yescarta^®^ brought in $264M (US dollars) in sales in 2018, largely due to the need for better treatment for DLBCL [396]. Kymriah^®^, which was the first CAR-T to be approved, was limited to 2018 sales of $28M (US dollars), hampered by both manufacturing concerns and approval for only B-ALL until May 2018, when it was also approved for DLBCL [397]. Blincyto^®^, which is currently only approved for B-ALL, brought in $230M (US dollars) in 2018 [398], which demonstrates that at least in B-ALL, it is competitive market-wise with the CAR-T thus far. A significant caveat to this, however, is that Blincyto^®^ has been approved since 2014, so it has had a longer period to build its market share.

**Table 6:** Comparison of TRBAs and CAR-T cell platforms as therapeutics.

Properties	Therapeutic Approach		
	T cell Redirection with TRBAs	Autologous CAR-T or NK Cells	Allogeneic CAR-T or NK Cells (Projected)
Currently approved and marketed (as of 20 June 2019)	1; Blincyto^®^ (anti-CD19 × CD3 BiTE^®^)	2; Kymriah^®^ and Yescarta^®^, both CD19-targeting autologous CAR-Ts	None
Current indications covered	R/R B-ALL	DLBCL, R/R NHL, B-ALL	None
Structure	Bispecific antibodies that bind both a tumor antigen and CD3ε on T cells	T cells engineered with synthetic gene construct encoding scFv fused to linker and activation domains	T cells engineered with synthetic gene construct encoding scFv fused to linker and activation domains
Source and homogeneity of T cell component	Endogenous T cells; No homogeneity (i.e., all CD3^+^ T cells may be engaged)	Expanded and activated endogenous T cells; homogeneity depends on process used	Could be homogeneous CD8^+^ T-cells, depending on cell type and approach
Antibody	Short half-life vs long half-life formats	Currently, mostly scFvs; possible unfolding, aggregation, tonic signaling; need for better binding constructs	Currently mostly scFvs—possible unfolding, aggregation, tonic signaling; need for better binding constructs
T-cell signaling domain(s)	CD3ζ	CD3ζ + 4-1BB (or OX40) and/or CD28	CD3ζ + 4-1BB (or OX40) and/or CD28
PD-1 inhibition of CD28 activity	Likely significant issue; may need to co-dose with PD-1 inhibitor	Use of 4-1-BB signaling domain should alleviate	Use of 4-1-BB signaling domain should alleviate
Drug-like properties	“Off-the-shelf” drug	Must be engineered from patient’s T cells (2–4 week process)	Depends on cell type and construct
Dosing	Multiple dosing; short half-life formats may require continuous dosing via pumps	Single dose	Single dose; multiple dose potentially available if engineered to eliminate HLA
Route of administration	IV; possible subcutaneous for future candidates	IV only	IV only
Long-term persistence and memory	Short half-life – only as long as continuously infused; long half-life – typically measured in weeks	Yes, but variable; longer persistence correlated with activity	Unknown but likely to be similar to autologous T-cells
Immune synapse	Normal and concentric; normal detachment	Abnormal and multifocal; fast detachment	Expected to be similar to autologous CAR-T cells
T cell signals at synapse	Signals 1, 3	Signals 1, 2 (sometimes), 3	Expected to be similar to autologous CAR-T cells
Killing mechanisms	Perforin and granzyme; Secondary: cytokine modulation of TME [123]	Perforin and granzyme; Fas/FasL axis; Secondary: cytokine modulation of TME [123]	Expected to be similar to autologous CAR-T cells
Serial killing	Yes, similar to CTLs	Yes, faster than TRBAs an CTLs	Expected to be the same as autologous T cells
	None; related to dosing and half-life	Yes, in responders	Unknown but expected
Bystander killing of antigen-negative cells	Demonstrated, as long as antigen-negative cells were in direct contact with antigen-positive cells [381]	Demonstrated, as long as antigen-negative cells were in direct contact with antigen-positive cells [380]	Unknown but expected based on CAR-T results
Toxicity	CRS, neurotoxicity	Higher CRS and neurotoxicity than TRBAs	Unknown but expected
Ability to attack solid tumors	To be determined; early data are mixed but not encouraging	To be determined; early data are mixed but not encouraging	Potential based on TIL correlation data
Trafficking	Passive	Active but limited; can be engineered to match tumor needs	Active; possible to engineered to match tumor needs
Trafficking into CNS	Not demonstrated; Unlikely if BBB is intact [395]	Demonstrated trafficking into CNS [399]	Unknown but expected based on CAR-T results
Need for lymphodepletion prior to treatment	No	Yes	Yes
Technical risk	Moderate; many platforms are working well	High but may be manageable	Currently very high
Need for “kill switch” or turn-off methodology	No but nice to have, especially for long half-life formats	Moderate; nice to have	Very high; must have for safety
Accessibility	High–off-the-shelf biologic drug	Only available at specific medical centers thus far; 2–4 week process time before therapy	Projected to eventually have availability similar to biologic drugs
Cost of goods	Relatively low; Antibody-like or slightly higher depending on type of TRBA platform	Very high (more than a $75,000 process)	Projected to be low to medium once cell manufacturing process is established
Cost to patient/payers	Medium ($89,000/course; $178,000 for predicted two course therapy) *	Very high ($373,000 for treatment of DLBCL; $475,000 for Kymriah^®^ treatment of B-cell ALL) **	Projected as medium to high, depending on cell type and construct

Abbreviations: BBB, blood-brain barrier; B-ALL, B-cell acute lymphoblastic leukemia; BiTE^®^, bispecific T-cell engager; CAR-T cell, chimeric antigen receptor-T cell; CD, cluster of differentiation; CNS, central nervous system; CRS, cytokine release syndrome; DLBCL, diffuse large B-cell lymphoma; IV, intravenous; NHL, non-Hodgkin lymphoma; NK, natural killer (cells); R/R, relapsed or refractory; PD-1, programmed cell death protein 1; scFv, single chain, Fragment variable (antibodies); TIL, tumor-infiltrating lymphocytes; TRBA, T-cell redirecting bispecific antibody. * Quote is for the cost of two-course treatment with Blincyto^®^ [400]. ** Quotes for cost of CAR-T therapies [401].

The overall therapeutic strategy behind both CAR T-cells and TRBA is the same, that is, harness the cytotoxic function of T (or NK, NKT) cells to target and kill tumor cells, as well as to overcome the escape mechanisms utilized by the tumor cells. However, these two technologies differ significantly. TRBAs typically have a significantly higher affinity on the tumor antigen arm than on the T-cell arm, so once dosed, they will largely distribute to the site of the tumor antigen and coat the antigen-positive tumor cells. These coated cells then become targets for T cells that enter the tumor. Thus, TRBAs have a significant dependency on both the ability of cytotoxic T-cells to enter the tumors and for those cells to become activated upon binding. It has been shown, however, that TRBAs can even cause T-regulatory cells to kill tumors cells [402], so the forced formation of the synapse also serves to activate the T-cells. The activation signals for TRBAs are limited to CD3ζ and there is no provision for signal 2, at least in the current generation of TRBAs. Thus, TRBAs are potentially limited in the TME by the suppressive effects of PD-1/PD-L1 and similar regulatory immune checkpoints.

CAR-T cells, on the other hand already are armed with the antibodies and seek out tumor cells as a “ready-made killing machine.” Additionally, autologous CAR-T cells are activated ex vivo, typically by CD3, CD28, and/or cytokine treatment, providing a stimulant for killing tumor cells immediately upon dosing. Finally, when CAR-T cells bind to the target cells, the costimulatory domains activate the CAR-Ts further, giving them a significant edge over TRBAs in terms of killing potential.

As described in Section 4.1, another approach that falls in between CAR-Ts and TRBAs is the use of autologous T cells, activated and armed with a TRBA *ex vivo*, followed by reintroduction to the patient [21]. This approach is similar to CAR-Ts in the sense that autologous T-cells are used and activated ex vivo but more similar to TRBAs in the sense that the “drug” is a bispecific antibody conjugate. Also, in this case, there is no recombinant T-cell activation domain, so the activation rests solely with CD3ζ, thus missing one of the key components of a CAR-T strategy. This is probably one of the key reasons why the ex vivo T-cell/TRBA approach has garnered only limited interest.

As mentioned in Section 2.2, the potency of the CAR-T cells may come from the unusual multifocal synapses they form with the target cells, allowing for faster killing rates and quicker time to release [112,113]. Additionally, CAR-T cells possess their own ability to provide signal 2 in the intracellular activation domains (e.g., CD27, 4-1BB, OX40). Finally, as mentioned in Section 6.6.4., one potential checkpoint issue with natural T cells and bispecific antibodies is the ability of PD-1/PD-L1 interaction to override the CD28 activation signal [361]. In CAR-T cells possessing the 4-1BB signaling domain, which is becoming an increasing number due to the activation and proliferative signaling provided by 4-1BB as well as its ability to promote persistence and reduce T-cell exhaustion [138,403,404], this should not be an issue. Third generation CAR-Ts can include both CD28 and 4-1BB costimulatory domains (or other combinations; see Figure 3), so these newer CAR-T constructs should better promote both T-cell function, proliferation and persistence [134,404].

In both CAR T-cells and TRBA therapeutics, healthy and active T cells are a prerequisite. In most cancer patients, especially patients with hematological malignancies, multiple lines of therapy previously administered can severely impact the number and health of circulating T cells [313]. The impact of the patients T cell status will certainly limit the effectiveness of both CAR T-cells and TRBAs. However, during the process of generating a CAR T-cell product, the patients T-cells are removed and manipulated ex vivo during their genetic manipulation to express the CAR, allowing for recovery and expansion prior to dosing [313]. As a result, the impact of the pretreatment can be mitigated to some extent and less chance is factored into the overall success of the CAR T-cell approach, compared to TRBA approach. This will be even less of an issue for truly allogeneic CAR T-cell products in which healthy, highly characterized cells are used as the starting material.

Currently, TRBAs do have an advantage with regard to ease of manufacturing, consistent product and dose control. This is especially significant in situations in which multiple dosing is required or preferred or for tumor target antigens that are also expressed on normal tissues. The ability to titer the dose and control the exposure of the agent in vivo has a significant advantage when tight control of the therapeutic agent is required to limit on-target, off-tumor toxicity. In contrast, currently used autologous CAR T-cell therapeutics are usually only dosed once (or at most, twice), owing to the challenges associated with current manufacturing technologies and the products themselves are a heterogeneous mixture of different T-cell subtypes with varying CAR expression.

### 9.2. Clinical Comparison of TRBAs vs. CAR-T Cells

T-cell redirecting therapeutics, including both TRBAs and autologous CAR-Ts, have revolutionized the treatment of hematological malignancies over the past half-dozen years or so. These forms of therapy, as described in this review, have significant promise for use and success in many forms of cancer. As shown in Table 4 and Table 6, two autologous CAR-T cell therapies, Kymriah^®^ and Yescarta^®^, were approved by the US-FDA in 2017, just a few months apart. Kymriah^®^ has now been approved for treatment of B-cell ALL (August 2017) and R/R DLBCL (May 2018), whereas Yescarta^®^ has been approved for R/R DLBCL and PMBCL (Oct 2017). Similarly, Blincyto^®^ was approved in 2014 for Philadelphia-negative chromosome B-cell ALL and has more recently (July 2017) tacked on approval for Philadelphia-chromosome-positive B-cell ALL. The CD19-targeting CAR, Lisocabtagene maraleucel (Liso-cell; JCAR017) and BCMA-targeting bb2121 are currently in Phase 3 clinical trials for DLBCL and multiple myeloma, respectively, so it would be expected that they may be the next T-cell redirected biologics in line for consideration for marketing approval.

Table 7 provides a glimpse at a few example clinical trial outcomes into the current status of these lead T-cell redirecting biologics. Key results that have enthused clinicians and patients are objective response rates (ORR) of up to ~80% and complete responses (CRs) above 50% for CAR-T treatments of the aggressive B-cell lymphoma, DLBCL. Median duration time for responses in DLBCL have been over 10 months for the CAR-Ts and 7 months for Blincyto^®^ (Table 7). Some of these responses in DLBCL have been durable, while others have faded over time. Kymriah^®^ has been impressive for the treatment of B-cell ALL, with CRs above 80%, as compared with Blincyto^®^, which at least for Philadelphia chromosome-negative B-ALL, CRs were above 30% (Table 7).

It is now well known that persistence of the CAR-Ts is highly critical for their success and is correlated with responses [413]. This is borne out by data provided in clinical trials with Kymriah^®^. For B-cell ALL patients who responded to the treatment, the geometric mean half-life for the CAR-T cells was ca. 22 days; for non-responders, the half-life was only 2.7 days [290]. The time to the last detectable CAR-Ts was 170 days for responders versus only about 29 days for non-responders [290]. Similarly, for DLBCL responding patients treated with Kymriah^®^, the half-life was calculated to be about 91 days for responders versus 15.4 days for non-responder patients. In this case, times to the last detectable CAR-Ts in circulation were 289 days for responders versus 57 days for non-responders [290]. There are two take-aways from these data: first, the persistence of the same CAR-T was significantly different between the two different patient populations, indicating that disease-specific factors play a role in persistence of autologous CAR-T cells; second, it is clear that longer CAR-T persistence in both disease types was correlated with responders. As we learn more about what differentiates responders from non-responders, we may be able to influence the level of persistence of CAR-Ts in patients to improve response rates.

While the data for anti-BCMA treatment of MM by CAR-Ts and TRBAs is in its infancy, a few results recently cited from early clinical trials give hope for future MM treatment paradigms. The very high ORR (85%) and CR (45%) associated with bb2121 treatment of MM (Table 7) is very encouraging, as are the data with the anti-BCMA TRBA, AMG420, which are from a Phase I ascending dose paradigm (i.e., several of the patients dosed were dosed at below therapeutic levels, which skews the numbers downward). At the projected optimal dose of 400 µg/day, there were four CRs [411].

The real goals of clinical treatment are durable, complete responses. Currently, both TRBAs and CAR-Ts have high initial response rates but have a significant relapse rate. Thus, both increasing the number of patients with CRs and extending the durability through CAR-T persistence and improved design of TRBAs will be critical to clinical improvements. While it appears thus far that CAR-T cell therapy is more potent that TRBA therapy, this analysis comes with huge caveats, including the very small sample size, the fact that the only TRBA included in the analysis is Blincyto^®^, which due to its short half-life must be dosed continuously and the overall toxicity profile, which appears to be higher for CAR-Ts than for TRBAs. Moreover, it is still too early to pass judgement on the various forms of T-cell redirected therapies, as so many new variables have been tested over the past few years that we really are just now beginning to learn the critical quality attributes for each type of therapeutic.

It has been suggested that for TRBAs to compete with future CAR-Ts, CRs will need to be >50% with durations longer than a year, with progression-free survival and overall survival of 12 and 18 months, respectively and cure rates of at least 30%. Those kinds of data, however, particularly extending to treatments beyond B-ALL and DLBCL, will make virtually any TRBA or CAR-T highly competitive.

There are many reasons for failure of either a TRBA or CAR-T. Target antigen loss accounts for about a third of all TRBA and CAR-T failures. For example, in six clinical trials using CD19 CAR-T cells to treat B-ALL, the relapse rates ranged from 29–57% [414]. Of those relapses, 7–25% were due to loss of the targeted antigen, CD19, on the tumor cells [414]. Related to antigen loss is antigen down-regulation, which may change the copy number of the tumor-associated antigen from high to low and perhaps even as low or lower than copy number on normal tissues, which presents a significant challenge with respect to therapeutic window. Another major reason for treatment failure is modification of the tumor antigen on the surface of cells due to mutations, many of which have lost the targeted epitope [415] and possibly also alternative expression of splice variants. Antigen down-regulation and modification are thought to make up a second third of treatment failures. The final major factor resulting in failures is treatment-related toxicities. While this is being controlled by treatment with drugs to limit CRS and by changes to dosing paradigm, it is still a significant factor in failure.

### 9.3. Future Improvements

#### 9.3.1. TRBAs

There are several improvements that may be made to increase TRBA efficacy and reduce toxicity, many of which have been described in this paper. Some of these include greater emphasis on targeting epitopes close to the membrane that allow the greatest level of T-cell activation [221,224,226], greater emphasis on geometry of both binding arms that allows for strongest synapse formation and killing potency (c.f., Figure 8 [17]) and improvement in understanding and applying affinity to both the CD3ε and tumor antigen arms. With respect to affinity, it has been often considered that the affinity between the CD3ε arm should be lower than the affinity of the second arm to the tumor antigen. This allows for distribution to the tumor and coating of the tumor antigen-positive tumor cells [416]. Too high of an affinity on the CD3ε arm of a TRBA can potentially lead to distribution into T-cell rich tissues such as lymph node and spleen rather than into tumor tissues [416] and may potentially lead to toxicity [417]. One rule of thumb, which may or may not bear out with time, is to have at least a ten-fold higher affinity to the tumor antigen than to the CD3ε. This makes sense, since the goal is to have the TRBA distribute to the tumor, coat antigen-positive tumor cells and attract T-cells to those TRBA-coated tumor cells. Another approach to increasing the apparent affinity to the tumor versus T-cells is to increase the number of tumor antigen binding arms while keeping only a single CD3ε binding arm [227,228]. The 2:1 TCB platform recently described by Roche does exactly that, with a concomitant improvement in activity [227]. Intriguing designs for TRBAs in the future may include multiple tumor antigen binding arms with only a single CD3ε binding arm, such as the IGM Biosciences IGM-based TRBA, which has 10 binding arms for the tumor antigen and a single binding arm for CD3ε) [228].

Getting the right TRBA dose is a significant issue. Because TRBAs are so potent and even low doses can lead to significant CRS in some cases, the FDA carried out a study on clinical stage TRBAs concerning first-in-human dosing and concluded that initial doses needed to follow careful MABEL (minimum anticipated biological effect level) calculations to ensure safe dose escalation in the clinic [418]. Additional improvements in TRBAs may be more related to dosing paradigms, including step-up dosing when necessary to prevent or reduce the chance of CRS [390]. Another issue with TRBA dosing is the potential to overdose, which can lead to separate coating of T-cells and tumor cells in a manner that they will not interact, which would lead to a lack of efficacy [223]. Additionally, it is well known that continuous stimulation of T cells can lead to T-cell anergy, so TRBA dosing paradigms may be designed to provide oscillations in serum concentrations, which may decrease T-cell anergy [27]. Finally, since TRBAs do not provide a signal 2, combinations of TRBAs and either checkpoint inhibitors or possibly activators may increase the efficacy and therapeutic window.

The most important improvement in TRBAs would be to develop a TRBA format that either significantly limits or eliminates CRS, since CRS appears to be dose-limiting in most cases. A very recent publication highlighted the development of a new TRBA format that appears, at least in vitro and in animal experiments, to limit CRS significantly [417]. This TRBA consisted of a Fab arm binding to CD3ε on one side and two domain antibodies binding the tumor antigen in place of the other Fab arm [417]. Of critical importance, though, was the fact that the correlation of high potency and low CRS was largely due to the properties of the CD3ε-binding arm, F2B, which binds the CD3δε heterodimer at about 34 nM but does not measurably bind the CD3γε heterodimer [417]. Well-studied anti-CD3ε mAbs such as OKT3 bind both the CD3δε and CD3γε heterodimers at relatively high affinity, indicating that the mAb F2B epitope must be unique, which may also offer a unique signaling pattern [417]. Unfortunately, the CD3δε-specific F2B mAb does not cross-react with cynomolgus monkey CD3ε [417], so that will make preclinical toxicology more difficult to assess. Hopefully, additional TRBA platforms with improved binding arms, geometry, and/or affinity/avidity will be found that improve potency with concomitant low CRS.

#### 9.3.2. CARs

For CAR-based cell therapy designs, there are so many potential improvements that could be made, it is difficult to define which are the most important. Clearly, if a stable, off-the-shelf allogeneic CAR-T or CAR-NKT cell line with a defined PK/PD profile could be established that rivals the clinical results demonstrated by the approved autologous CAR-Ts, Kymriah^®^ and Yescarta^®^, that would be perhaps the most significant advance. This type of CAR would allow for broader distribution, immediate use as a therapeutic and hopefully, a less costly therapy. Additionally, such a cell line could be engineered in all the ways mentioned in this paper, including making it PD-1 or PD-L1-negative [146,362,363,364,365], engineering in the ability to produce antibodies in the local TME environment [143,144,145], adding in cytokine expression to help activation and proliferation [99,339,369,370], kill switches to control any adverse circumstances [98,301,302,303,341,342,343,344,348,349,350] and cloning in chemokine receptors to help in trafficking the cells to tumors [100,337,355,358,360].

As shown in Table 4 and Table 7, the different CAR-T therapeutics are constructed with different spacers, transmembrane domains and costimulatory domains. Virtually all CAR-T constructs today utilized the CD3ζ signaling domain, so that is a constant. Additionally, as mentioned previously, affinity, epitope and type of antibody used may have a significant impact on activity. A signaling analysis recently carried out suggested that co-stimulation with CD28 resulted in faster and higher downstream protein phosphorylation which correlated with effector T-cell function [419]. On the other hand, co-stimulation with 4-1BB was more correlated with genes associated with T-cell memory and as previously noted, also correlates with sustained activity and persistence [134,138,403,404]. The importance of one or the other profile may change with cell types, cancer types and indications, so it is too early to state broadly that one costimulatory form is preferable over another.

The appropriate affinity for a CAR has been a topic of considerable discussion. Early CARs were constructed with whatever antibodies were already available, many of them of mouse origin, as previously discussed. As CAR design has become more sophisticated, both the affinity [404,420] and target antigen epitope [421] of the CAR have become more critically designed. It has been noted that higher affinity CARs may interfere with serial killing and persistence, as well as potentially promoting T cell exhaustion or even activation-induced cell death [111]. In some cases, lower affinity CARs were correlated with greater cancer cell killing and tumor clearance [422] and may help to promote a faster off-rate from the tumor cell [112,113], which itself correlates with increased cytokine and chemokine expression [423]. Lower CAR affinity, however, can result in the requirement for higher target numbers to achieve activation, which is potentially great for differentiating normal tissue with low antigen expression from antigen-over-expressing cancer tissue but can limit tumor killing when target antigen is decreased by either lower expression [111,128] or through trogocytosis [130]. In fact, the strategy of lowering the CAR affinity to reduce on-target but off-tumor toxicity (i.e., binding to tissues with lower target antigen numbers (has been used to limit toxicities associated with targeting solid tumors) [420]. Thus, a key to improvement of future CARS will be the balancing of CAR affinity with the application of activation and signaling domains. A recent publication demonstrated that lowering the affinity of the CAR, coupled with the combination of both CD28 and 4-1BB signaling, produced a CAR with balanced and potentially optimal affinity/signaling properties [404].

As has been shown with TRBAs (Section 3.4) [15,17,221,224,226], it is also believed that membrane proximal epitopes on targeted antigens provide the best activity for CARs [421]. More distal epitopes might not be sufficient to generate a strong immunological synapse [224,424]. However, other considerations are equally important and not as implied based on sequence of the target antigen. For example, the “neighborhood” of the target antigen and how it can interfere with epitope access, as well as, its structural conformation can also interfere with the interaction of the TRBA interaction [425]. Thus, understanding the activity of the CAR in the presence of primary target cells, which may possess the most realistic target neighborhood, may be of significant benefit over cell lines into which the target antigen has been cloned for expression.

Changes in CAR construction not involving either the target-binding scFv or the signaling and/or costimulatory domains also can have a huge impact on CAR function and safety. A recent result suggests that minor changes can have enormous impacts on the performance and safety of a CAR-T cell. Ying et al. [426] generated and compared a series of modified CAR-T cells based on CTL019 (Kymriah^®^; Tisagenlecleucel-T) in which they altered the length of the CD8α transmembrane and hinge used. They found that one of their constructs, CD19-BBz(86), which possessed a 10 amino acid residue longer extracellular hinge as well as a 4 amino acid longer intracellular domain, was far superior to CTL019 in CRS and other functions [426]. This construct was recently taken into a Phase 1 clinical trial [427], demonstrating significantly superior safety (very low CRS and neurotoxicity) over most other clinical stage CAR-Ts and slower proliferation, while retaining high functionality (54.5% CRS obtained with B-cell lymphoma patients) [426]. This study demonstrates how even minor changes to “seemingly non-critical components” of the CAR can yield significantly improved CAR-Ts.

Once the CAR T-cell product is dosed, the expansion and persistence are completely under the control of the biology and state of the patient and the CAR T-cells. This loss of control is often the driver behind the toxic CRS and neurotoxicity observed in CAR T-cell therapeutics and has been forced CAR T-cell developers to design safety switch technologies and other management strategies to control these products once dosed [428,429]. Control of PK/PD and persistence of CAR-T products would be a significant advance. It is well recognized that CAR-T persistence is correlated with response [395]. This is exemplified by data from clinical trials with Kymriah^®^, in which the investigators were able to identify a clear correlation between patient population and pharmacokinetic properties of the CAR-T [290]. There were two distinct populations of patients, that is, responding patients in which the geometric mean half-life of the CAR-T was over 20 days and the time to last measurable CAR-T cells was 170 days and non-responding patients, in which the geometric mean of the CAR-T in circulation was less than three days and the CAR-Ts were gone within a month [290]. Understanding what biological signals or circumstances that differentiate these two populations might allow us to turn non-responders into responders, which could make the CAR-T therapeutics significantly more effective than they already are.

Similar to TRBAs, CAR-Ts suffer from the influences of two main biological facts: The potential negative influences of the tumor microenvironment of the T-cells, especially for solid tumors; and our ability to identify truly tumor specific target antigens. There are many negative influences on the biological responses to T-cells including the upregulation of check-point molecules like PD-1/PD-L1 and PD-L2, expression of suppressive cytokines like VEGF and TGF-β, the infiltration of myeloid-derived suppressive cells, tumor associated macrophages, cancer associated fibroblasts and regulatory T-cells, poor vascularization and hypoxic conditions [425]. Although we are just beginning to understand some of the factors driving these barriers, our ability to manipulate them will be key to overcoming them. With so many different pathways contributing to this physiochemical barrier, it is difficult to envision that a single agent therapeutic will be able to overcome them all and allow for the immune cells to have an effect. Consequently, TRBAs will no doubt have the most difficult time as they are only designed to address one aspect of the therapeutic strategy of localizing and activating T-cells near the tumor cells. The simplicity of these agents loses some of its appeal when one considers the need to now address many additional pathways that will need to be manipulated in order to achieve therapeutic responses. CAR T-cells, on the other hand, have built-in components that can be manipulated to address many, if not all, of these barriers. As previously discussed, the generation of a CAR T-cell involves genetic engineering to insert the CAR construct into the T-cell genome. It is entirely feasible to add additional modifications, even in a single genetic manipulation step, to address some of these barriers. For example, CAR T-cells can be engineered to express antibody fragments that will block the action of suppressive cytokines or check-point molecules. They can be stimulated ex vivo to upregulate chemokine receptors or other receptors that drive tumor localization [430].

Efforts to generate allogeneic CAR T-cell products will undoubtedly be highly engineered to not only address the allo-reactivity but also many of these barriers. Future allogeneic CAR-based cell products that are expected to be more homogenous and well characterized will be a significant improvement over current autologous CAR products with regard to understanding dose-response relationships. However, it still remains to be seen if even with these highly controlled allogeneic products they will be more predictable once dosed.

## 10. Summary and Future State

This is an exciting time for T-cell redirected therapeutics, both for protein-based bispecifics and cell-based CAR therapeutics. Both formats have shown significant glimpses of promise but also many shortcomings, several of which may be able to be addressed with engineering. With so many different types of TRBAs (Table 2) and CAR-Ts (Table 3 and Table 4) being studied in clinical trials today, it will be years before we understand the optimal structures and/or constructs with critical quality attributes that best address each particular type of cancer or indication. The differences between the two platforms, CAR-Ts and TRBAs, today is enormous, with ease of use, availability and costs favoring TRBAs where sheer potency driving CRs and duration of response currently favoring CAR-Ts. If allogeneic CAR-Ts become more widely available in easily engineered formats, this could significantly change the outlook, as the “off-the-shelf” CAR-Ts will then start to take on more of the characteristics of regular biologic drugs, that is, dosing and redosing, no need to wait for processing, lower costs and availability. This review has documented many or the ways in which the molecules and/or cells can be engineered to potentially increase the therapeutic window. Many of these engineered modifications or addons will be much easier to accomplish by engineering cells rather than protein biologics, so in the long-run, there does appear to be a potentially higher upside potential for CAR-Ts than TRBAs but that remains to be seen. The key to both formats is the ability to increase the therapeutic window by significantly decreasing both CRS-related and neuro-related toxicity, while maintaining or even increasing potency.

## Figures and Tables

**Table 1:** Overview of T-cell and NK-cell redirected therapeutics in clinical trials *.

Type	Clinical Stage			Total
	Phase I/II	Phase III	Approved	
T-cell or NK cell-redirecting bispecific Abs	59	0	2 **	61
Autologous CAR-T, CAR-NK, CAR-NKT cells	207	2	2	211
Allogeneic CAR-T, CAR-NK, CAR-NKT cells	14	0	0	14
Allogeneic NK or Autologous T cells engineered with Fc RIIIa for binding therapeutic antibodies	3	0	0	3
Total of CAR-T, T-cell or NK cell redirected killing of tumor cells	283	2	4	289

* BiStro Biotech Consulting LLC database, locked 20 June 2019. Data obtained from Clinicaltrials.gov, literature papers, company websites, analyst reports and other sources. ** One of these, Removab^®^, was voluntarily discontinued in 2017 by the sponsor.

**Table 2:** Bispecific antibody T- and NK-cell redirecting antibody formats represented in clinical trials *^,^**.

Bispecific T- or NK-Cell Redirecting Antibody Format ***	Clinical Stage			Total
	Phase I/II	Phase III	Approved	
Short half-life bivalent fragments (e.g., BiTE®s, DART®s, ImTACs, other bivalent fragments)	15	0	1	16
Half-life extended bivalent fragments (e.g., DART®-Fc, Extended half-life BiTE®s, TriTAC)	11	0	0	11
Asymmetric bivalent IgG-like (e.g., Trion, BEAT, Xencor H/A platform, Duobodies, other asymmetric platforms)	21	0	1 ****	22
Roche TCB 2:1, Chugai ART-Ig®-scFv and Teneobio 2:1 platforms (two binding sites for target cell, one for CD3)	4	0	0	4
ADAPTIR® and TandAb platforms (tetravalent platforms)	4	0	0	4
Chemically conjugated IgGs (tetravalent; two IgGs)	4	0	0	4
Total	59	0	2	61

Abbreviations: ADAPTIR, modular protein technology; ART-Ig^®^, asymmetric re-engineering technology–immunoglobulin; BEAT, bispecific engagement by antibodies based on the T cell receptor; BiTE^®^, bispecific T cell engager; DART^®^, dual affinity retargeting (antibody); ImmTAC, immune-mobilizing monoclonal TCR against cancer; TandAb, tandem diabody; TCB, T-cell bispecific; TriTAC, Trispecific T cell activating construct. * BiStro Biotech Consulting LLC database, locked 20 June 2019. Data obtained from Clinicaltrials.gov, literature papers, company websites, analyst reports and other sources. ** Out of a total known 122 bispecific antibodies being studied in clinical trials as of 20 June 2019. *** The platforms and abbreviations are described in the text and in Figure 7. **** Voluntarily removed from marketing in 2017.

**Table 3:** CAR-T formats *.

CAR-T Format	Clinical Stage			Total
	Phase I/II	Phase III	Approved	
Autologous CAR-T **—Single CAR	176	2	2	180
Autologous CAR-T **—Multiple CARs for different targets or multiple CAR-Ts dosed in combination	30	0	0	30
Autologous CAR-NKT	1	0	0	1
Allogeneic CAR-T	11	0	0	11
Allogeneic CAR-NK or -NKT	3	0	0	3
Total	221	2	2	225

* BiStro Biotech Consulting LLC database, locked 20 June 2019. Data obtained from Clinicaltrials.gov, literature papers, company websites, analyst reports and other sources. ** As far as can be ascertained from public documents, all of these appear to be based on αβ-T cells.

**Table 5:** Targets of clinical stage T-cell redirected therapeutics, TRBAs and CARs *^,^**.

Primary Target	Primary Indications	Therapeutic Format			Total
		TRBAs	CAR-T/NKs	rCells Expressing FcγRIIIa	
CD19	B-cell cancer (NHL, etc.)	2	88	0	90
BCMA	MM	7	26	1	34
CD123	AML	5	8	0	13
Mesothelin	Solid tumors	1	12	0	13
GD2	Solid and neurological tumors	2	10	0	12
CD20	B-cell cancer (NHL, etc.)	5	4	2	11
CD33	AML	6	4	0	10
HER2	Solid tumors	3	6	0	9
CD22	B-cell cancer (NHL, etc.)	0	8	0	8
CD30	HL	1	5		6
PSMA	Solid tumor (prostate)	4	2	0	6
EGFRvIII	Neurological tumors	2	4	0	6
EGFR	Solid tumors	1	3	0	4
CD38	MM	2	2	0	4
EpCAM	Solid tumors	2	2	0	4
PSCA	Solid tumor (prostate)	1	3	0	4
CEA (CEACAM5)	Solid tumors	2	1	0	3
HIV	Virus	1	1	0	2
Glypican-3	Solid tumors	1	1	0	2
Flt3	AML	1	1	0	2
NKG2D ligands	Solid tumors	0	2	0	2
Claudin 18.2	Solid tumors	0	2	0	2
DLL3	SCLC	1	1	0	2
CS1 (SLAMF7)	MM	0	2	0	2
MUC16	Solid tumors	1	1	0	2
Lewis-Y	Solid tumors	0	2	0	2
cMet	Solid tumors	0	2	0	2
Others with single candidate	Mostly solid tumors	10	16	0	26
Undisclosed/other	Unknown	0	6	0	6
Total	--	61	225	3	289

* BiStro Biotech Consulting LLC database, locked 20 June 2019. Data obtained from Clinicaltrials.gov, literature papers, company websites, analyst reports and other sources. ** Abbreviations: AML, adult acute myeloid leukemia; BCMA, B-cell maturation antigen; CAR, chimeric antigen receptor; CD, cluster of differentiation; CEA, carcinoembryonic antigen; EGFR, epidermal growth factor receptor; EpCAM, epithelial cellular adhesion molecule; GD2, disialoganglioside antigen; HER2, human epidermal growth factor receptor; HL, Hodgkin’s lymphoma; MM, multiple myeloma; NHL, non-Hodgkin lymphoma; NK, natural killer (cells); NKG2D, natural killer group 2D; PD-L1, programmed death ligand-1; PSCA, prostate stem cell antigen; PSMA, prostate-specific membrane antigen; rCells, recombinant NK or T-cells; SCLC, small cell lung cancer; TRBA, T-cell redirecting bispecific antibody.

**Table 7:** Examples of clinical data with T-cell redirecting biologics.

Property	T-Cell Redirecting Biologic Drug							
	Kymriah^®^	Blincyto^®^	Kymriah^®^	Yescarta^®^	Liso-cel	Blincyto^®^	bb2121	AMG420
Sponsor	Novartis	Amgen	Novartis	Gilead (Kite)	Juno/Celgene	Amgen	Bluebird/Celgene	Amgen
Format	CAR-T; 4-1BB CS	TRBA (BiTE^®^)	CAR-T; 4-1BB CS	CAR-T; CD28 CS	CAR-T; 4-1BB CS	TRBA (BiTE^®^)	CAR-T; 4-1BB CS	TRBA (BiTE^®^)
Trial	EL	TW	JU	ZU	TC	Phase 1	Phase 1	Phase 1
Target	CD19	CD19	CD19	CD19	CD19	CD19	BCMA	BCMA
Indication	B-ALL	B-ALL (PCN)	DLBCL	DLBCL	DLBCL	DLBCL	MM	MM
# Number of Patients	63	271	93	101	73	11	33	42
ORR	ND	ND	52%	83%	80%	55%	85%	31%
CR/CR *	83%	34%	40%	58%	59%	36%	45%	17%
PR	20%	ND	12%	25%	21%	18%	39%	10%
Median response duration time	NR	7.7 mo	11.7 mo	11.1 mo	10.2 mo NR	13.3 mo	11.8 mo	NR
Grade 3+ AEs	ND	ND	89%	98%	16%	90%	ND	ND
CRS incidence	77%	15%	58%	58%	37%	ND	76%	38%
Grade 3+ CRS	ND	nk	22%	11%	1%	ND	6%	ND
Neurotoxicity	ND	65%	21%	64%	25%	71%	ND	ND
Grade 3+ Neurotoxicity	ND	13%	12%	32%	15%	20%	ND	0%
Elimination half-life	21.7 d RP; 2.7 d NRP	NA	91.3 d RP; 15.4 d NRP	ND	ND	NA	ND	NA
References	[290]	[384,405]	[290,406]	[291,407]	[408]	[393,409]	[295,410]	[411,412]

* Abbreviations: B-ALL, B-cell acute lymphoblastic leukemia; BCMA, B-cell maturation antigen; CD, cluster of differentiation; BiTE^®^, bispecific T-cell engager (short half-life, continuously infused); d, days; CAR-T, chimeric antigen receptor T-cell; CS, co-stimulation domain; DLBCL, diffuse large B-cell lymphoma; EL, ELIANA trial; JU, JULIET trial; Liso-cel, Lisocabtagene maraleucel (JCAR017); MM, multiple myeloma; mo, months; NA, not applicable; ND, no data; NR, not reached (during testing period covered); NRP, non-responding patients; PCN, Philadelphia chromosome-negative; RP, responding patients; TC, TRANSCEND-CORE; TRBA, T-cell redirecting bispecific antibody; TW, Tower trial; ZU, ZUMA-1 trial. #: Number.

## Abbreviations

AML	adult acute myeloid leukemia
APC	antigen presenting cell
ART-Ig	asymmetric re-engineering technology—immunoglobulin
ATTACK	asymmetric tandem trimerbody for T cell activation and cancer killing
B-ALL	B cell acute lymphoblastic leukemia
BBB	blood-brain barrier
BEAT	bispecific engagement by antibodies based on the T cell receptor
BiKE	bispecific killer engager
BiTE	bispecific T-cell engager
CAR	chimeric antigen receptor
CD	cluster of differentiation
CIKs	cytokine-induced killers
CLL	chronic lymphocytic leukemia
CNS	central nervous system
CR	complete response
CRS	cytokine released syndrome
CTL	cytotoxic T lymphocytes
DART	dual affinity retargeting (antibody)
DLBCL	diffuse large B cell lymphoma
DVD-Ig	dual variable domain immunoglobulins
EGFRt	truncated version of epidermal growth factor receptor
EMA	European Medicines Agency
EpCAM	epithelial cell adhesion molecule
Fc	fragment, crystallizable
FIT-Ig	Fabs-in-tandem immunoglobulins
GVH, GVHD	graft-versus-host (disease)
HC	heavy chain
HLA	human leukocyte antigen
HSV-TK	herpes simplex virus thymidine kinase
iCasp9	inducible caspase-9
Ig	immunoglobulin
ImmTAC	immune-mobilizing monoclonal TCR against cancer
iNKT	invariant NKT (cells)
ITAM	immunoreceptor tyrosine activation motif
KIH	knobs-into-holes
LC	light chain
MATH	mutant-allele tumor heterogeneity
MHC	major histocompatibility complex
MM	multiple myeloma
NK	natural killer (cell)
NKG2D	natural killer group 2D
NKT	natural killer T (cell)
NSCLC	non-small cell lung cancer
OR	objective response
PBMCs	peripheral blood mononuclear cells
pMHC	peptide-MHC complex
PR	partial response
R/R	relapsed/refractory
sCAR	switchable chimeric antigen receptor
scFv	single chain, fragment variable
SEED	strand exchange engineered domain
SMAC	supramolecular activation cluster
TandAb	tandem diabody
TBE	target cell-biologic-effector cell (complex)
TCB	T-cell bispecifics
TCR	T-cell receptor
TILs	tumor infiltrating lymphocytes
TME	tumor microenvironment
TRBA	T-cell redirecting bispecific antibody
TriKE	trispecific killer engager
TITAC	trispecific T cell activating construct
US-FDA	United States Food and Drug Administration
